# Specific DNAzymes cleave the 300–618 nt of 5′UTR to inhibit DHAV-1 translation and replication

**DOI:** 10.3389/fmicb.2022.1064612

**Published:** 2022-12-12

**Authors:** Yanglin Li, Ling Wei, Anchun Cheng, Mingshu Wang, Xumin Ou, Sai Mao, Bin Tian, Qiao Yang, Ying Wu, Shaqiu Zhang, Juan Huang, Qun Gao, Di Sun, Xinxin Zhao, Renyong Jia, Mafeng Liu, Dekang Zhu, Shun Chen, Yanling Yu, Ling Zhang, Leichang Pan

**Affiliations:** ^1^Institute of Preventive Veterinary Medicine, Sichuan Agricultural University, Chengdu, China; ^2^Key Laboratory of Animal Disease and Human Health of Sichuan Province, Sichuan Agricultural University, Chengdu, China; ^3^Avian Disease Research Center, College of Veterinary Medicine, Sichuan Agricultural University, Chengdu, China

**Keywords:** duck hepatitis A virus type 1, the 300–618 nt of 5′UTR, DNAzyme, DZ454, cleavage, inhibition

## Abstract

DNAzymes effectively inhibit the expression of viral genes. Duck hepatitis A virus type-1 (DHAV-1) genomic RNA carries an internal ribosome entry site (IRES). The IRES initiates the translation of DHAV-1 *via* a mechanism that differs from that of cap-dependent translation. Therefore, it is an attractive target for the treatment of DHAV-1. In this study, we designed 6 DNAzymes (Dzs) specifically targeting 300–618 nt sequence in the DHAV-1 5′untranslated region (UTR; a predicted IRES-like element). In the presence of divalent metal ions, three designed DNAzymes (DZ369, DZ454, and DZ514) efficiently cleaved the 300–618 nt sequence of the DHAV-1 5′UTR RNA. The activity of the Dzs was particularly dependent on Mg^2+^ ions. Subsequently, the translation inhibitory activity of these Dzs was determined by western blotting experiments. The Dzs effectively inhibited the translation mediated by the 300–618 nt of DHAV-1 5′UTR in duck embryo fibroblasts (DEFs). Importantly, DZ454 showed the strongest inhibitory effect, and its inhibition was time and dose dependent. However, none of the Dzs showed significant inhibition of cap-dependent translation. These results suggest that these Dzs show specificity for target RNA. Moreover, DZ454 inhibited the replication of DHAV-1. In conclusion, the designed DNAzymes can be used as inhibitors of DHAV-1 RNA translation and replication, providing new insights useful for the development of anti-DHAV-1 drugs.

## Introduction

Duck hepatitis A virus type-1 (DHAV-1) is an acute and highly lethal pathogen in ducklings that is found worldwide (Xie et al., 2018). DHAV-1 belongs to the *Picornaviridae* family (Jiang et al., 2014). Its genome comprises a 7,700-nt single-stranded positive-sense RNA sequence with an open reading frame (ORF) encoding 2,249 amino acids, a 5′ untranslated region (5′UTR), a 3′ untranslated region (3′UTR), and a poly (A) tail (Kim et al., 2006; Ding and Zhang, 2007). These untranslated regions play important roles in both viral translation and replication (Chen et al., 2018; Liu Y. et al., 2020). The ORF is first translated into a polyprotein, which is cleaved into multiple viral proteins. These viral proteins play important roles in the life cycle of DHAV-1 and manipulate certain physiological and biochemical reactions in the host cells (Cao et al., 2016; Zhang et al., 2017; Yang et al., 2018; Lai et al., 2019; Sun et al., 2019; Liu Z. et al., 2021; Li J. et al., 2022; Li X. et al., 2022; Liu et al., 2022).

The 5′UTR of picornavirus genomes contains internal ribosome entry sites (IRESs), which are classified into 5 main types based on distinct genome sequences, secondary structures, and function modes (Jang et al., 1988; Sweeney et al., 2012; Asnani et al., 2015). The IRES of the DHAV genome belongs to a type IV IRES (Pan et al., 2012). IRES-mediated translation initiation of viral mRNAs differs from cap-dependent translation initiation of most eukaryotic mRNAs (Leppek et al., 2018). Similar to other picornaviruses, DHAV-1 can overcome the massive cellular translation block after infection, and the mechanism driven by IRES provides advantages for viral protein synthesis (Han et al., 2021; Liu Y. et al., 2021). The activity of IRESs depends on specific viral RNA motifs, with each type sharing a common RNA structural core (Martinez-Salas et al., 2017). Moreover, the evolution of IRESs is mainly induced by intratypic recombination and is independent of other parts of the genome (Kirkegaard and Baltimore, 1986; Arhab et al., 2020). With these features, IRESs play important roles in viral virulence and have become attractive targets for designing antiviral drugs.

Currently, many compounds are used in gene therapy, including antisense oligonucleotides (Laxton et al., 2011), short interfering RNAs (siRNAs; Kanda et al., 2007; Ma et al., 2014), ribozymes (Mao et al., 2014), DNAzymes (Hou et al., 2006), etc. Compared with other compounds, DNAzymes are characterized by lower molecular weight, more stable structures, higher catalytic activity, easier modification and labeling, and lower immunogenicity (Zhou et al., 2017). Based on these advantages, DNAzymes have been applied to detection of various viral infections, such as the homogeneous detection of enterovirus EV71 and CVB3 infections (Du et al., 2021). Since they were discovered in the 1990s, DNAzymes have been reported to cleave RNA molecules into multiple segments (Santoro and Joyce, 1997; Liu et al., 2017). Divalent metal ions enhance the activity of DNAzymes (Li et al., 2020). DNAzyme not only has the cleavage effect of ribozyme, but also has the antisense inhibitory effect of antisense nucleotide (Zhou et al., 2017). To date, a variety of viruses have been reported to be cleaved and inhibited by DNAzymes. Roy et al. (2008), Robaldo et al. (2014) and Kumar et al. (2009) found that DNAzymes specifically cleave hepatitis C virus (HCV) RNA and inhibit HCV RNA translation and replication. Hou et al. (2006) reported that DNAzymes inhibited the expression of the hepatitis B virus X gene. Singh et al. (2012) and Sugiyama et al. (2011) discovered that DNAzymes inhibited the expression of the HIV-1 integrase gene and the replication of HIV. Taken together, DNAzymes can regulate viral translation and replication, which may lead to a new therapeutic direction for diseases caused by RNA viruses.

In this study, we explored the effect of DNAzymes on the 300–618 nt region of the DHAV-1 5′UTR. Our results showed that DNAzymes specifically cleaved 300–618 nt of the DHAV-1 5′UTR, thereby inhibiting the translation and replication of DHAV-1. These results are expected to provide theoretical support for gene therapy of duck viral hepatitis.

## Materials and methods

### Cells and viruses

The primary duck embryo fibroblasts (DEFs) were extracted from specific pathogen-free 9- to 11-day-old duck embryos and cultured in minimum essential medium (MEM; Sangon Biotech) containing 10% new-born calf serum (NCS; Millipore) and incubated at 37°C with 5% CO_2_ in an incubator.

The virulent DHAV-1 X strain (GenBank: JQ316452.1) was provided by the Institute of Preventive Veterinary Medicine, Sichuan Agricultural University. The virus titer was measured *via* 50% tissue culture infective dose (TCID_50_) assays. DEFs were infected with DHAV-1 (100 TCID_50_) for 2 h, and unbound virus was removed by washing twice with phosphate-buffered saline (PBS) before the cells were overlaid with MEM containing 2% NCS.

### Bioinformatics analysis

The nucleotide identities of the 5′UTR in the DHAV-1 X strain (GenBank: JQ316452.1) and the IRES elements in other viruses (all belonging to the type IV IRES family), such as Hepatitis C virus (HCV, GenBank: AB016785), porcine teschovirus-1 (PTV-1, GenBank: AB038528) and duck hepatitis A virus type-3 C-GY (DHAV-3, GenBank: EU352805), were analyzed by DNASTAR.

According to the above homology comparison results, DHAV-1 5′UTR 300-618 nt may be DHAV-1 IRES element. And the secondary structure of the 300–618 nt DHAV-1 5′UTR was predicted using the M-FOLD program.

### DNAzyme synthesis

**Figure 1:**
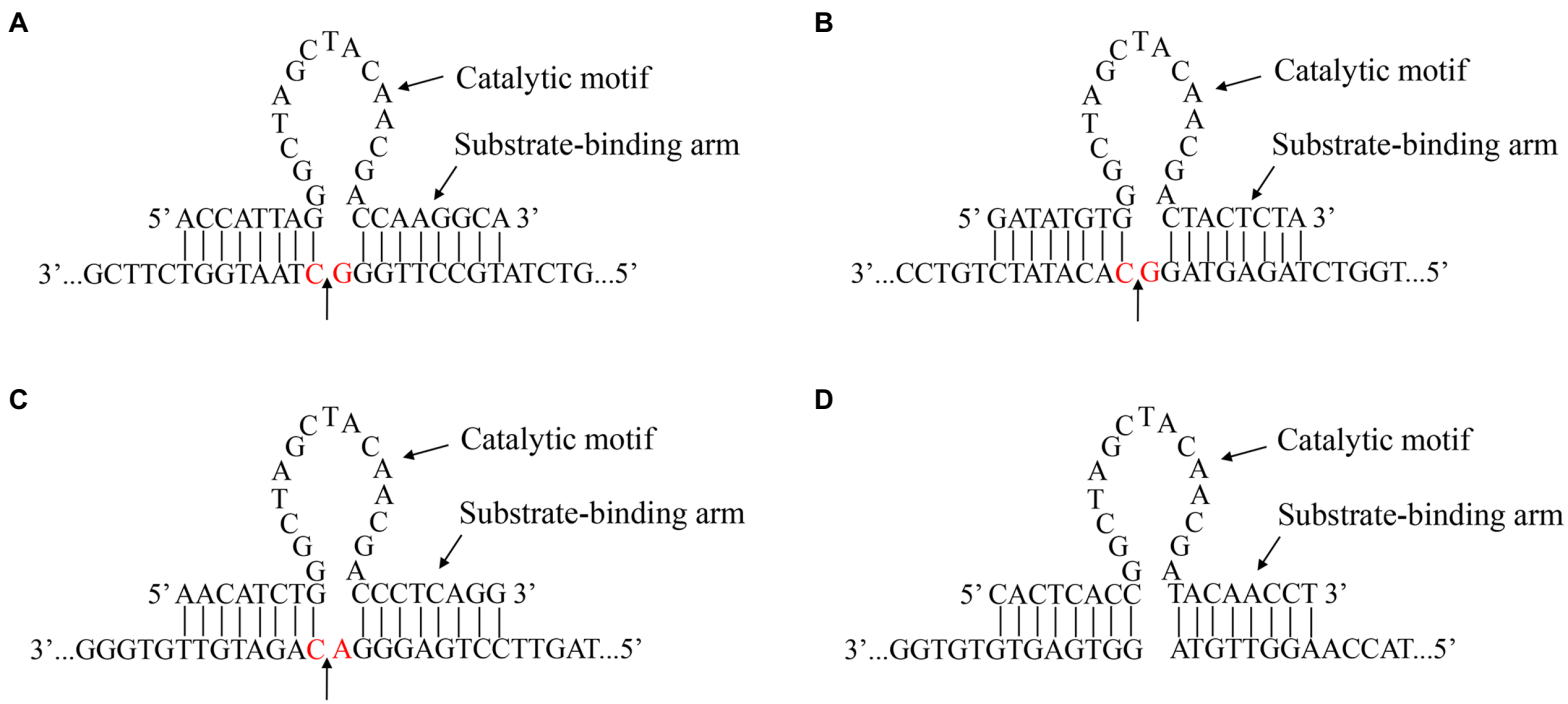
Gene sequence and secondary structure of the DNAzymes. The recognition and catalytic sequences of DZ369 **(A)**, DZ454 **(B)**, DZ514 **(C)**, and DZ000 **(D)**, and their corresponding cleavage sites. The red base shows the location of a target site.

On the basis of the 300–618 nt DHAV-1 5′UTR, we designed five 10–23 DNAzymes, namely, DZ369, DZ454, DZ514, DZ454-7, and DZ454-9, that targeted different regions (Table 1). Each of these DNAzymes carried a conserved 15 nt (5′-GGCTAGCTACAACGA-3′) catalytic motif. The catalytic motif was flanked by substrate-binding arms, and the substrate-binding arms specifically recognized the target RNA (Figure 1). Moreover, DZ000, which carried the same catalytic motif but lacked the cleavage site of the 300–618 nt region of the DHAV-1 5′UTR, was used as the negative control (Figure 1).

**Table 1:** Sequences of DNAzymes.

Name	Sequence	Flank length	Location of the target site
DZ369	ACCATTAGGGCTAGCTACAACGACCAAGGCA	8 + 8	369-370(G-C)
DZ454	GATATGTGGGCTAGCTACAACGACTACTCTA	8 + 8	454-455(G-C)
DZ514	AACATCTGGGCTAGCTACAACGACCCTCAGG	8 + 8	514-515(A-C)
DZ454-7	ATATGTGGGCTAGCTACAACGACTACTC	7 + 7	454-455(G-C)
DZ454-9	AGATATGTGGGCTAGCTACAACGACTACTCTAG	9 + 9	454-455(G-C)
DZ000	CACTCACCGGCTAGCTACAACGATACGAGCT	8 + 8	\

### Plasmid construction

To construct pGEM-T/IRES and pRed-IRES-EGFP plasmids, we designed primers based on the gene sequences of the DHAV-1 X strain and pDsRed-Express-C1. Total RNA was extracted from DEFs infected with the DHAV-1 X strain using RNAiso Plus Reagent (TaKaRa) according to the manufacturer’s instructions. Then, the total RNA was reverse transcribed into cDNA with a PrimeScript™ II 1st Strand cDNA Synthesis Kit (TaKaRa). The DHAV-1 IRES sequence was amplified from the cDNA *via* PCR and integrated into the pGEM-T and pEGFP-N1 vectors with a one-step cloning kit (Vazyme). Then, the Red sequence was amplified from the pDsRed-Express-C1 plasmid and integrated into a pEGFP-IRES-N1 vector with a kit.

### *In vitro* cleavage of DHAV-RNA with DNAzyme

The *in vitro* run-off transcript of the 300–618 nt DHAV-1 5′UTR RNA (361 nt, function as IRES element, related to DHAV-1 RNA translation) was established with a ScriptMAX Thermo T7 Transcription Kit. Next, 3 μl of DNAzyme (2 pmol/ml) and 3 μl of substrate RNA (2 pmol/ml) were added to buffer with 50 mmol/L Tris–HCl (pH = 7.5), 120 mmol/L NaCl and 10 mmol/L metal ions and incubated at 37°C for 30 min. Then, the samples were cooled on ice and mixed with 10 × RNA Loading Buffer, and the cleaved fragments were resolved *via* 5% agarose gel electrophoresis.

### Lactate dehydrogenase activity assay

To measure the cytotoxicity of each DNAzyme, DNAzymes (concentrations of 6, 8, and 10 μmol/L) were transfected into DEFs, and after 48 h, the cell supernatant was collected and an LDH Cytotoxicity Assay Kit (Beyotime) was used to measure LDH levels.

### Western blotting analysis

DEFs were cotransfected with the recombinant plasmid pRed-IRES-EGFP and DNAzymes. The cells were lysed in 100 μl of cell lysis buffer containing 1% PMSF (Beyotime). The cell lysate was centrifuged, and the supernatant was collected. Samples were fractionated by SDS–PAGE, transferred to PVDF membranes, and blocked with 5% nonfat dry milk at room temperature for 5–6 h. The membranes were incubated overnight at 4°C with primary antibodies diluted in blocking buffer. The membranes were washed three times with TBS-Tween and incubated for 1 h at 37°C with the respective secondary antibodies diluted in blocking buffer. The membranes were washed three times with TBS-Tween, and bound proteins were detected with an enhanced chemiluminescence (ECL) chromogenic kit (Beyotime).

### RNA extraction and viral RNA load in DEFs

Total RNA was isolated using RNAiso Plus Reagent (TaKaRa) according to the manufacturer’s instructions. Viral RNA levels were determined by RT–qPCR using a One Step PrimeScript RT–PCR kit (TaKaRa) on an Applied CFX96 Real-Time PCR detection system (Bio-Rad). The viral copy number was measured by the following method: standard RNA was diluted in a gradient of EASY Dilution Buffer. According to the sequence of DHAV-1 3D gene (GenBank: JQ316452.1), designed primers (P1: 5′-TGATGAGATATGGCAGGTAGAAGGA-3′; P2: 5′-CACGCAAGTTGATTCACAATAGA-3′) and a probe (FP: FAM-TGTGTTCAGGATCCCCATGTACTACCGTG-TAMRA) were used for this amplification. A gene copies/reaction ranging from 1 × 10^10^ to 1 × 10^5^ was used. A regression curve was constructed by plotting the threshold cycle (Ct) values versus the logarithm of the RNA copy number. Kinetic curves and standard curves were obtained with an iCycler IQ Detection System $\left(Y=-3.274X+39.955,R^{2}=0.998\right)$ . The viral copy number was obtained by substituting Ct values from samples into the equation representing the standard curve.

## Results

### Analysis and secondary structure prediction of the DHAV-1 5′UTR 300–618 nt sequence

**Figure 2:**
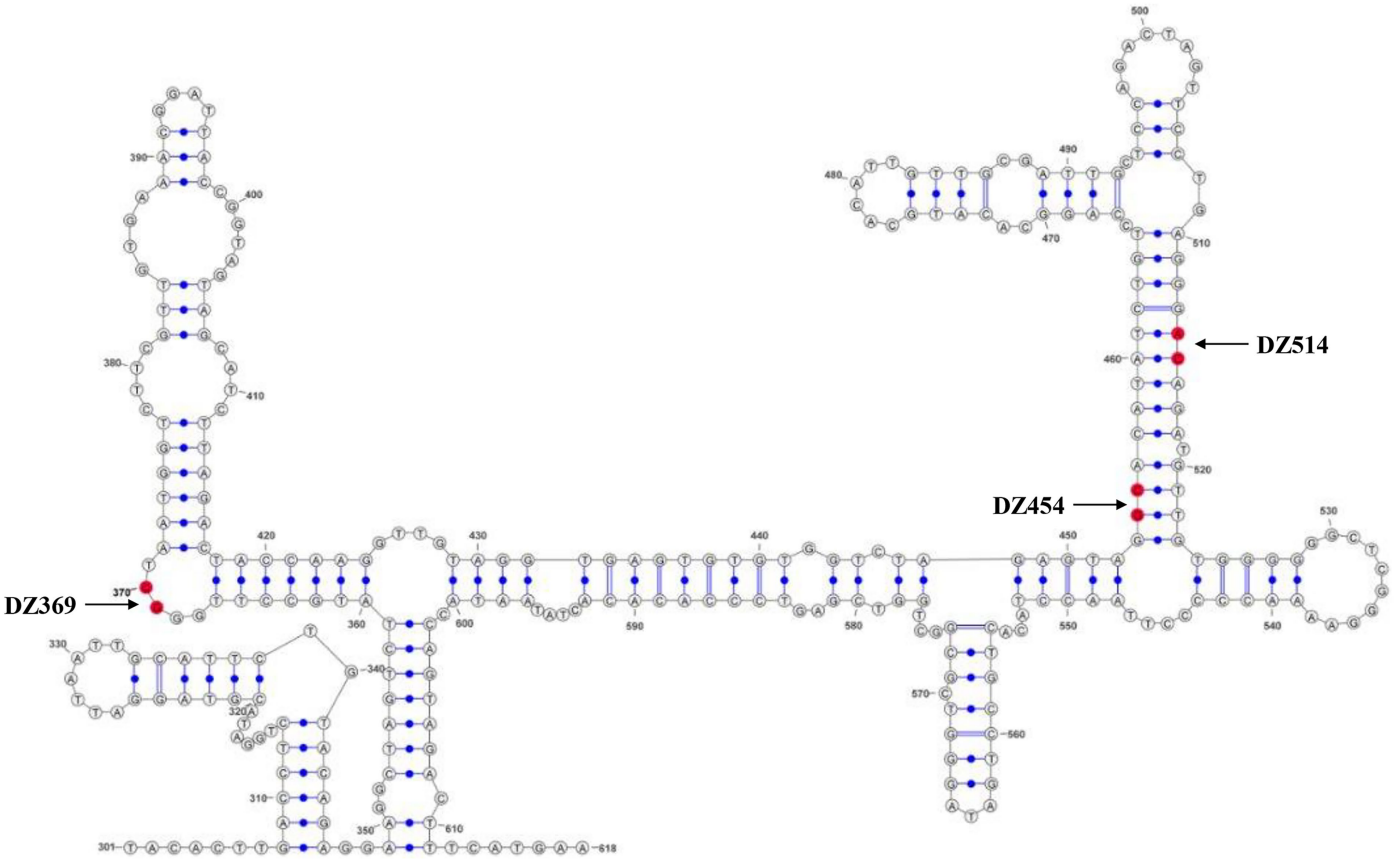
The secondary structure of the DHAV-1 5′UTR 300–618 nt sequence. The bases marked in red are the potential target sites of the DNAzymes.

Using DNASTAR software, we found that the 300–618 nt DHAV-1 5′UTR exhibited nucleotide identities (40.2% and 45.3%) similar to the sequences of the HCV IRES and PTV-1 IRES. Previous studies had compared the 5′UTR sequences of DHAVs and found that nucleotide identity was > or =94% with homologous serotypes and < or =73% with heterologous serotypes (Fu et al., 2008). Similarly, our analysis showed that the 5′UTR in DHAV-1 (X) showed only 69.6% identity with the DHAV-3 IRES (C-GY), suggesting differences between the heterologous serotypes. Then, we applied M-FOLD to model the secondary structure of the 300–618 nt of DHAV-1 5′UTR, and the cleavage sites for each Dz are shown by arrows (Figure 2).

### DNAzymes specifically cleave 300–618 nt of the DHAV-1 5′UTR

**Figure 3:**
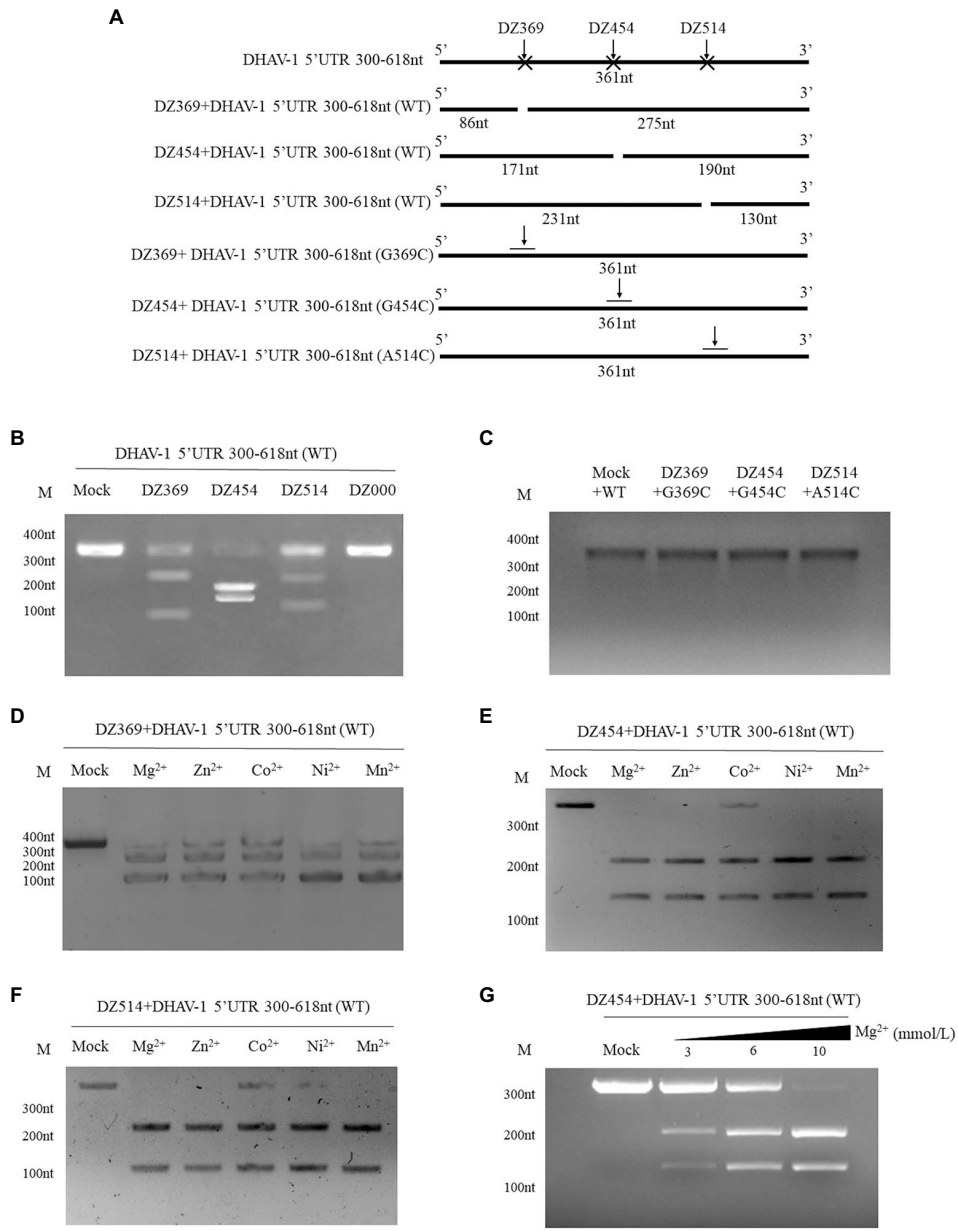
Different DNAzymes cleave the DHAV-1 5′UTR 300–618 nt sequence *in vitro*. **(A)** Schematic representations showing the Dz cleavage sites on the 300–618 nt sequence in the DHAV-1 5′ UTR and some mutants (G369C, G454C and A514C) associated with cleavage sites; the size of the cleaved fragment is shown. **(B)** In the presence of Mg^2+^, the 300–618 nt DHAV-1 5′UTR RNA (WT) reacted with various Dzs, and the cleaved fragments were analyzed *via* 5% agarose gel electrophoresis. **(C)** In the presence of Mg^2+^, the 300–618 nt DHAV-1 5′UTR RNA mutants reacted with various Dzs, and the fragments were analyzed *via* 5% agarose gel electrophoresis. **(D-F)** With the addition of Mg^2+^, Zn^2+^, Co^2+^, Ni^2+^, and Mn^2+^, DZ369, DZ454 and DZ514 cleaved target RNA *in vitro*, as analyzed *via* 5% agarose gel electrophoresis. **(G)** With the different concentrations of Mg^2+^and DZ454 cleaved target RNA *in vitro*, as analyzed *via* 5% agarose gel electrophoresis.

The 300–618 nt of the DHAV-1 5′UTR, a predicted IRES-like element, is related to DHAV-1 RNA translation. We designed several DNAzymes to inhibit its function. To evaluate the cleavage efficiency of various Dzs, *in vitro* cleavage reactions were performed. In the presence of Mg^2+^, DZ369, DZ454, and DZ514 exhibited significant cleavage activity *in vitro*. However, the control group DZ000 failed to cleave the 300–618 nt of DHAV-1 5′UTR RNA (Figures 3A,B). Then, some mutants associated with cleavage sites were constructed to further determine the expected sites (Figure 3A). The cleavage efficiency of DNAzymes on mutant was performed by *in vitro* cleavage reactions. As shown in Figure 3C, DNAzymes (DZ369, DZ454, and DZ514) failed to cleave the corresponding mutant.

Divalent metal ions can enhance the activity of DNAzymes. To further study the best metal ions to use with the designed Dzs, five common metal ions, Mg^2+^, Zn^2+^, Co^2+^, Ni^2+^, and Mn^2+^, were added to *in vitro* cleavage reaction mixtures. With the addition of various metal ions, DZ369 failed to completely cleave the 300–618 nt of DHAV-1 5′UTR RNA (target RNA) into two fragments (Figure 3D). DZ454 did not completely cleave the target RNA in the presence of Co^2+^, but it completely cleaved the target RNA into two nucleic acid fragments in the presence of the other metal ions (Figure 3E). Similarly, DZ514 failed to fully cleave the target RNA in the presence of Co^2+^ or Ni^2+^ (Figure 3F). Various metal ions facilitated Dz cleavage of the target RNA with different efficiencies. Among these ions, Mg^2+^ promoted the cleavage of these Dzs with the highest efficiency. Then, we wondered whether the promotion of Mg^2+^ is in a dose dependent manner. Different concentrations of Mg^2+^ were added to *in vitro* cleavage reaction mixtures. It was found that Mg^2+^ significantly enhanced the activity of DNAzyme in a dose-dependent manner (Figure 3G). These results indicate that Mg^2+^ may be the most effective cofactor for these Dzs.

### DNAzymes inhibit the translation mediated by 300–618 nt of DHAV-1 5′UTR

**Figure 4:**
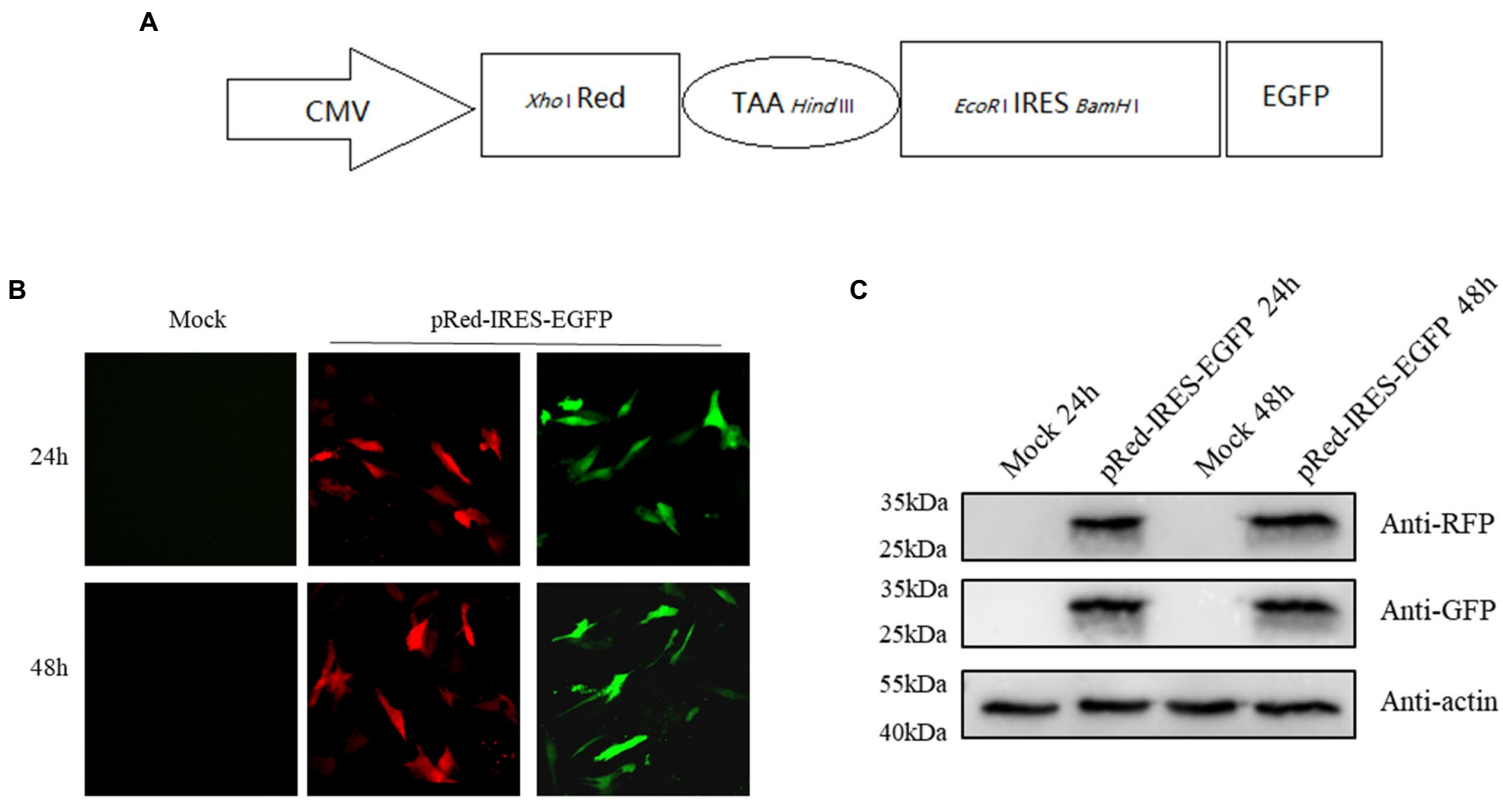
The DHAV-1 5′UTR 300–618 nt sequence initiates downstream gene expression. **(A)** Schematic representation showing the bicistronic vector pRed-IRES-EGFP. **(B)** DEFs were transfected with plasmids, and green fluorescence and red fluorescence were observed under a microscope 24 and 48 h after transfection. **(C)** The cells were harvested 24 and 48 h after transfection, and immunoblot analyses were performed with the indicated antibodies.

**Figure 5:**
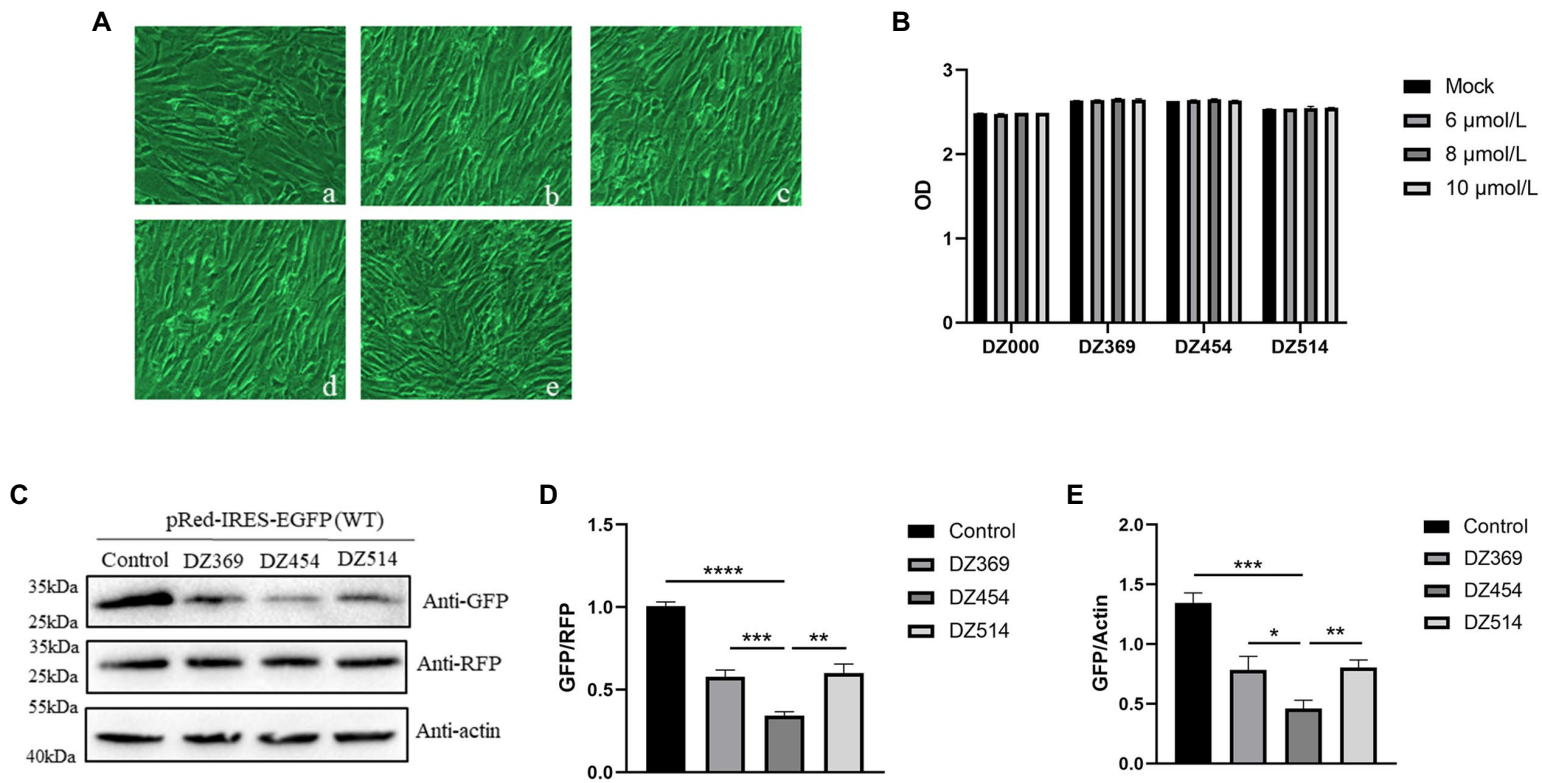
DNAzymes inhibit the translation mediated by the 300-618 nt of DHAV-1 5′UTR. **(A)** Cell morphology was observed 48 h after transfection, (a) Mock; (b) DZ000; (c) DZ369; (d) DZ454; and (e) DZ514. **(B)** The cell culture supernatant was collected for 48 h after transfection with Dzs, and the absorbance of the samples was measured. **(C)** pRed-IRES-EGFP was cotransfected with DZ369, DZ454, DZ514 or DZ000 (as the control group) in DEFs, and cells were harvested 48 h later and analyzed by immunoblotting with the indicated antibodies. **(D,E)** Band intensity was quantified with ImageJ software, and RFP or β-actin was used as the mandatory control or loading control. The ratio of GFP to RFP or GFP to β-actin was normalized to the respective levels in the control group. Differences between two groups were analyzed by Student’s *t*-test and were considered significant when *p < 0.05; **p < 0.01; ***p < 0.001; or ****p < 0.0001.

**Figure 6:**
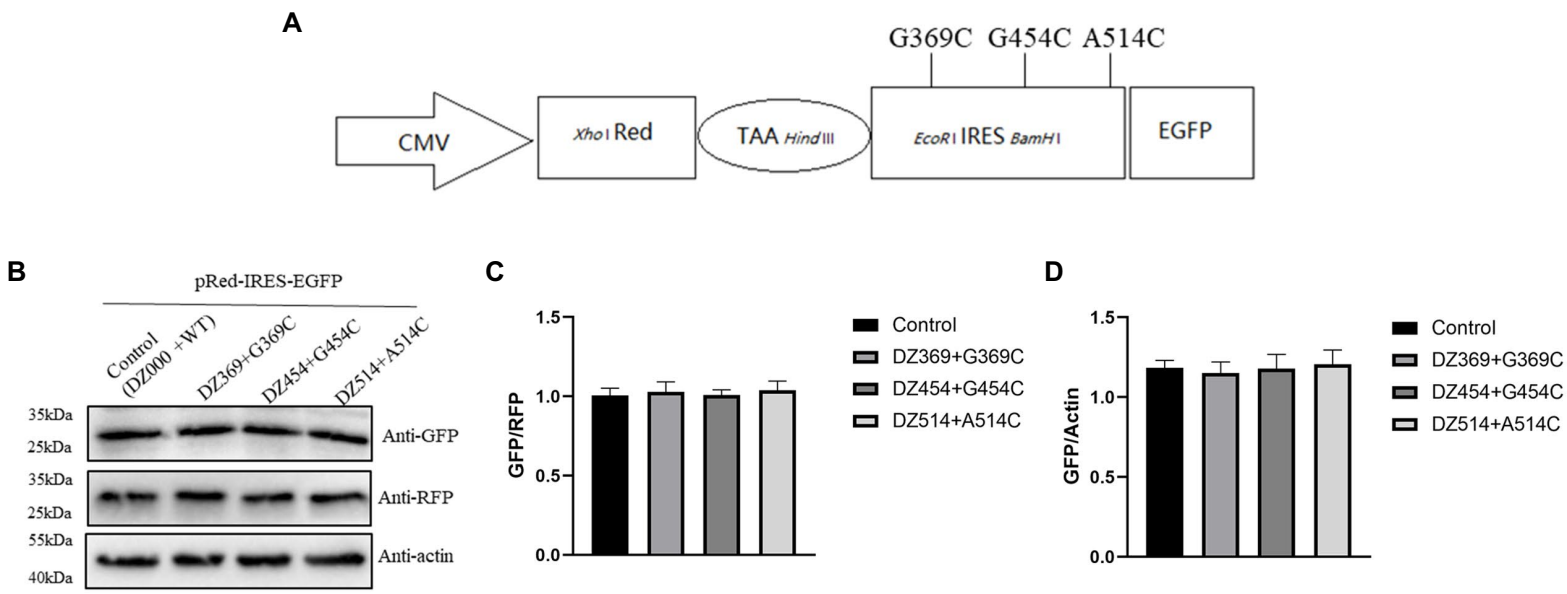
DNAzymes failed to inhibit the translation mediated by the 300–618 nt of DHAV-1 5′UTR mutants. **(A)** Schematic representation showing the pRed-IRES-EGFP mutants. **(B)** pRed-IRES-EGFP mutant was cotransfected with DZ369, DZ454, DZ514 or DZ000 (as the control group) in DEFs, and cells were harvested 48 h later and analyzed by immunoblotting with the indicated antibodies. **(C,D)** Band intensity was quantified with ImageJ software, and RFP or β-actin was used as the mandatory control or loading control. The ratio of GFP to RFP or GFP to β-actin was normalized to the respective levels in the control group.

To evaluate the effect of DNAzymes on cap-dependent translation and DHAV-1 IRES-mediated translation, a bicistronic vector was constructed on the basis of the 300–618 nt DHAV-1 5′UTR (Figure 4A). We transfected pRed-IRES-EGFP plasmids into DEFs and observed the expression of fluorescent proteins under a fluorescence microscope (Figure 4B). In addition, the expression levels of RFP and EGFP were measured by western blotting (Figure 4C). The results indicated that the constructed bicistronic vector was successfully expressed. Then, we cotransfected pRed-IRES-EGFP and Dzs into DEFs and 48 h later measured the expression of EGFP. We found that all three DNAzymes (DZ369, DZ454, and DZ514) exerted significant inhibitory effects on translation mediated by the 300–618 nt of the DHAV-1 5′UTR. DZ454 showed the strongest inhibition (Figures 5C-E). However, these molecules did not show inhibitory effects on cap-dependent translation (Figure 5C). Meanwhile, we constructed some pRed-IRES-EGFP mutants to determine the expected cleavage sites of Dzs (Figure 6A). We found that all three DNAzymes (DZ369, DZ454, and DZ514) did not show inhibitory effects on translation mediated by the DHAV-1 5′UTR 300-618 nt mutants (Figures 6B-D). Importantly, the results of cell morphology and LDH activity experiments showed that none of the DNAzymes were cytotoxic to the DEFs (Figures 5A,B).

**Figure 7:**
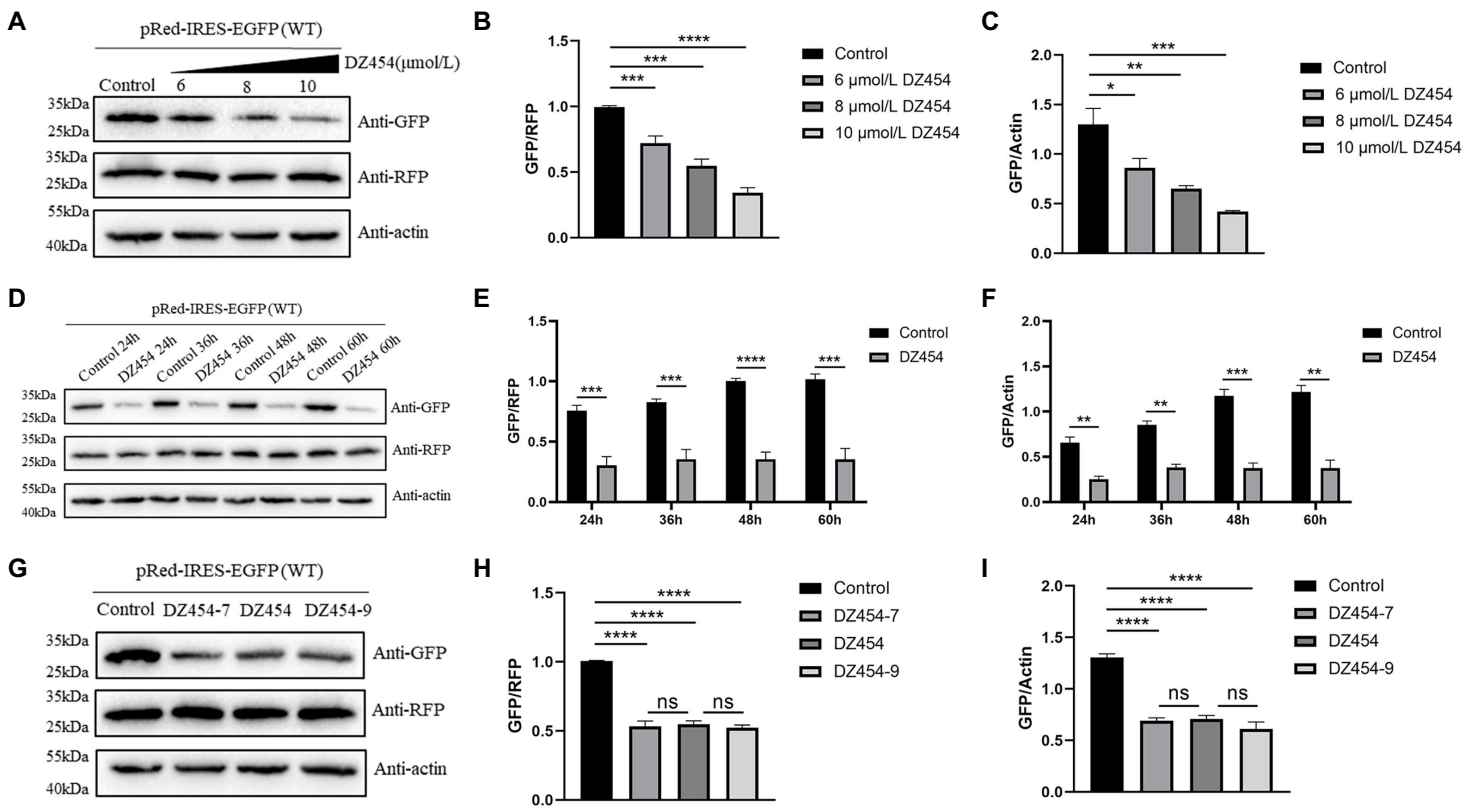
DZ454 inhibits the translation mediated by the 300–618 nt of DHAV-1 5′UTR. **(A)** pRed-IRES-EGFP was cotransfected with 6, 8, and 10 μmol/L DZ454 in DEFs, and 48 h later, the cells were collected and analyzed by immunoblotting with the indicated antibodies. **(D)** pRed-IRES-EGFP was cotransfected into DEFs with 10 μmol/L DZ454, and the cells were harvested at different time points for immunoblot analysis with the indicated antibodies. **(G)** pRed-IRES-EGFP and DZ454 of different flank lengths (DZ454-7, DZ454, or DZ454-9) were cotransfected into DEFs. The cells were collected 48 h after transfection, and western blot analysis was performed with the indicated antibodies. **(B,E,H)** Band intensity was quantified using ImageJ software, and RFP was used as the mandatory control. The ratio of GFP to RFP was normalized to the control group. **(C,F,I)** Band intensity was quantified using ImageJ, and β-actin was used as the loading control. The ratio of GFP to β-actin was normalized to the level in the control group. Differences between two groups were calculated by Student’s *t*-test and significance is indicated by *p < 0.05; **p < 0.01; ***p < 0.001; or ****p < 0.0001.

Considering these results, we wondered whether the inhibition of translation is time-dependent or dose-dependent. Different concentrations of DZ454 and pRed-IRES-EGFP were cotransfected into the DEFs, and the expression of EGFP was detected 48 h later. Compared with that of the control group, the inhibitory effect was found to be significant and dose-dependent effect (Figures 7A–C). Then, transient cotransfection experiments were performed using pRed-IRES-EGFP and 10 μmol/L DZ454 in DEFs, and the expression of EGFP was measured at different time points after transfection. Compared with that in the control group, the expression of EGFP in the DZ454 group was significantly inhibited at all time points, and the inhibition was increasingly obvious with prolonged time (Figures 7D–F). The results showed that the inhibition of DZ454 on the DHAV-1 5′UTR 300–618 nt sequence activity was closely related to the concentration of DZ454 and the interaction time.

The substrate-binding arms on both sides of the catalytic motif were generally composed of 7–9 oligonucleotides. To explore whether the flank length of DNAzyme affects its inhibitory effect, we designed and synthesized DZ454, DZ454-7, and DZ454-9. We cotransfected pRed-IRES-EGFP and 10 μmol/L DZ454, DZ454-7, or DZ454-9 into DEFs and detected the expression of EGFP after 48 h. We found that no significant difference in the expression of EGFP among the three DZ454 groups (Figures 7G–I). These results indicate that changes in the DNAzyme flank length did not affect its catalytic activity.

Taken together, these results demonstrated that DNAzymes specifically inhibited translation initiation mediated by the 300–618 nt region of the DHAV-1 5′UTR.

### DZ454 negatively regulates DHAV-1 replication in DEFs

**Figure 8:**
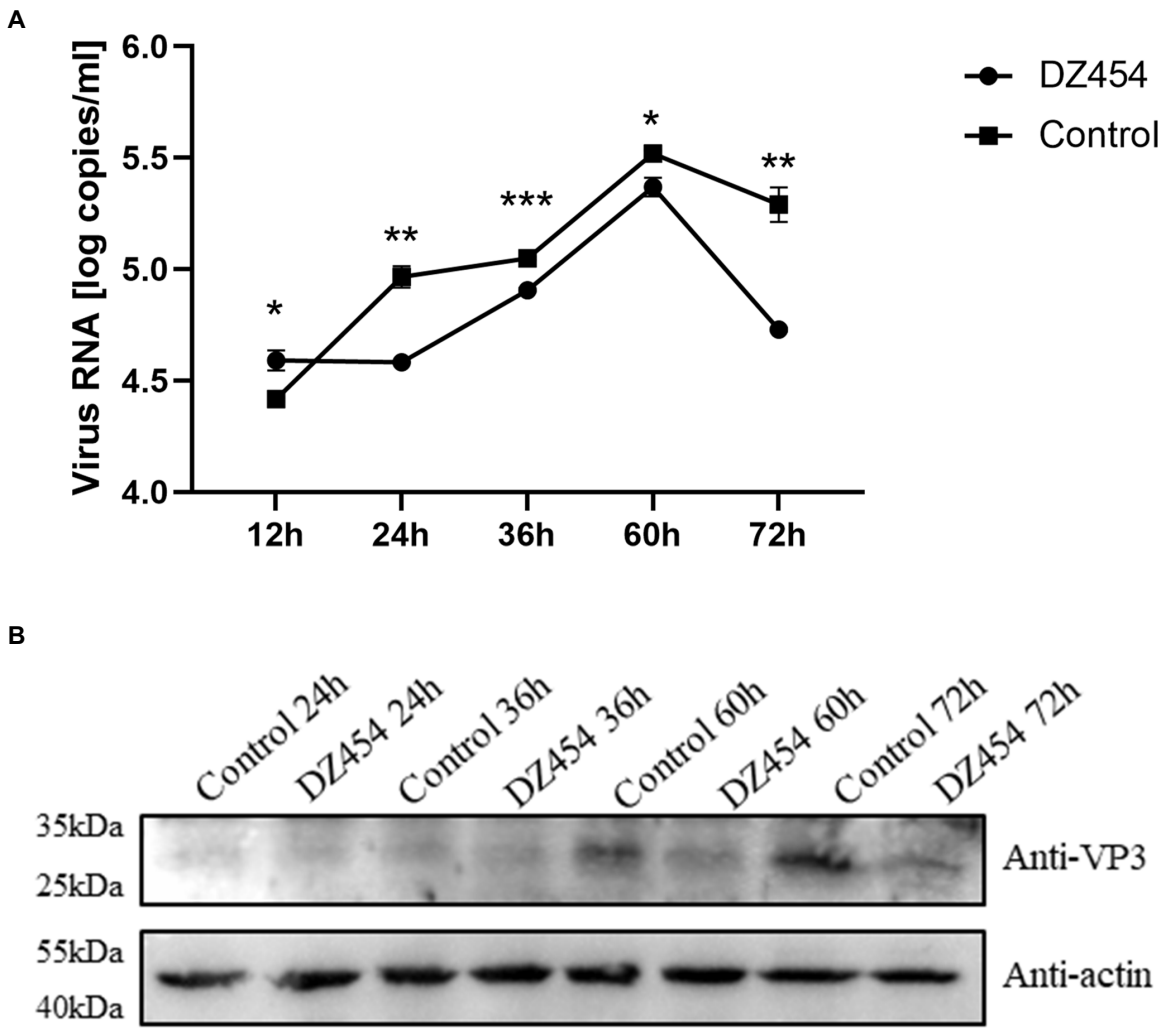
DZ454 inhibits the replication of DHAV-1. **(A)** DZ454 was transfected into DEFs, and the cells were infected with 100 TCID_50_ of the DHAV-1 X strain. RT-qPCR was used to measure virus copy numbers after 12, 24, 36, 60, and 72 h. The difference between the DZ454 group and the control group was analyzed by Student’s *t*-test; *p < 0.05; **p < 0.01; or ***p < 0.001. **(B)** The structural protein VP3 was detected in DEFs transfected with DZ454.

These above showed that DZ454 inhibited the activity of the DHAV-1 5′UTR 300–618 nt sequence. Next, we wondered whether it affects DHAV-1 replication. Therefore, we transfected 10 μmol/L DZ454 into DEFs infected with 100 TCID_50_ of the DHAV-1 X strain and determined the viral copy number after 12, 24, 36, 60, and 72 h by quantitative PCR (the sequence of DHAV-1 3D gene). Compared with that in the control group, the viral copy number in the DZ454 group decreased after 24 h, indicating that DZ454 exerted a significant inhibitory effect. The degree of inhibition peaked at 72 h (Figure 8A). Meanwhile, we detected VP3 expression at different time points after DZ454 transfection. Compared with that in the control group, the VP3 protein expression levels in the DZ454 group were significantly reduced (Figure 8B).

## Discussion

Since the discovery of the IRES, the regulation of IRES-mediated translation has been regarded as a critical step for picornavirus infection, with important effects on virulence, tissue tropism, and pathogenicity (Liu W. et al., 2020). Previous studies have shown that DNAzymes target IRESs of specific RNA viruses and inhibit their activities (Roy et al., 2008; Kumar et al., 2009; Laxton et al., 2011; Singh et al., 2012; Robaldo et al., 2014). DNAzymes cleave purine-pyrimidine regions within a RNA virus genome (Zhou et al., 2017). Many sites satisfy this condition in the 300–618 nt DHAV-1 5′UTR, and we randomly selected three sites. Although all base sequences identified by DZ369, DZ454, and DZ514 were cleavage targets, the cleavage activities of these three Dzs differed (Figure 3). These differences may have been related to a variety of factors: at cleavage sites, different spatial structures of each site and different GC contents around the site; in Dzs, the gene sequence and secondary structure of the Dzs differ; in target RNA, the secondary structure is unpredictable. In addition to three DNAzymes (DZ369, DZ454 and DZ514) described herein, other DNAzymes may exhibit better cleavage activity.

Metal ions are essential elements for the catalytic activity of most known DNAzymes and play key roles in the catalytic process (McGhee et al., 2017; Rosenbach et al., 2020). We found that DNAzymes showed different cleavage activity rates in the presence of divalent metal ions (Figures 3D–F), which may have been related to the inherent phosphate affinities of the different metal ions (Schnabl and Sigel, 2010).

The IRES in DHAV-1 are located between 300 and 618 nt of its 5′UTR, which is an approximate range. The actual length of the DHAV IRES may be longer or shorter. To verify its length, it will be necessary to perform site-directed mutagenesis and back mutation and then assess the functional activity of each part.

In this study, we discovered that DZ454 inhibited the translation mediated by the 300–618 nt of DHAV-1 5′UTR more strongly than DZ369 or DZ514 in DEFs (Figure 5). The results reported by Singh et al. (2012) showed that, although the inhibition rate of DIN116 was 75%, the inhibition effect of DIN54 reached only 20% in the same cells. In different cells, the inhibitory effect of the same DNAzyme on the same gene also profoundly differed. DZ454 exerted the strongest inhibition on translation mediated by the 300–618 nt of the DHAV-1 5′UTR in DEFs, while DZ514 showed the strongest inhibition in DF-1 (Supplementary Figure S1). The difference in DNAzyme activity may be related to the various steric hindrance effects of different cleavage sites, and the different content of divalent metal ions in different cells. An analysis of different flank lengths indicated that DNAzyme was more likely to inhibit the activity of target RNA by cleaving the phosphodiester linkage at the target site (Figure 7G).

The inhibition of DHAV-1 RNA replication by DNAzyme is another explanation for the inhibitory effect of DNAzyme on DHAV-1 IRES. The inhibitory effect of DNAzyme may occur during the viral replication period or the intermittent viral replication period. The increase and decrease in viral copy number reflects a dynamic variable not a dynamic equilibrium; therefore, the increase in DHVA-1 RNA does not follow a linear trend (Figure 8A).

Although DZ369, DZ454, and DZ514 can inhibit the expression of EGFP, the inhibitory effect is not ideal and, in theory, is expected to be stronger. The possible reasons for the unexpected findings are different steric hindrances that affect the cleavage effect; different target sites that lead to different cleavage efficiencies; target site secondary structures that may mask cleavage sites; etc. It has been reported that the activity of the DNAzyme with flanking sequences modified by LNA was much higher than that of an unmodified DNAzyme (Vester et al., 2002; Fahmy and Khachigian, 2004). The stability of a DNAzyme was enhanced by the use of a 3′-3′-inverted thymidine, phosphorothioate linkages, and 2′-O-methyl RNA (Schubert et al., 2003). Therefore, further studies are needed to determine which modifications can improve the cleavage efficiency without affecting the stability of the designed DNAzymes.

In conclusion, we confirmed that the designed DNAzymes inhibited the translation activity of the DHAV-1 5′UTR 300–618 nt sequence by specifically cleaving it, with DZ454 showing the strongest inhibitory effect on translation. DZ454 also exerted an inhibitory effect on viral replication. Our study broadens the knowledge of interactions between DNAzyme and IRES structural elements, which may lead to a potential approach for the development of anti-DHAV-1 drugs.

## Data availability statement

The original contributions presented in the study are included in the article/Supplementary material, further inquiries can be directed to the corresponding author.

## Ethics statement

The animal study was reviewed and approved by the Institutional Animal Care and Use Committee of Sichuan Agriculture University (Protocol Permit Number: SYXK (川) 2019-187).

## Author contributions

YL and LW conceived, designed, and wrote the manuscript. AC modified the manuscript. MW conceived and supervised the study. XO, SM, BT, QY, YW, SZ, JH, QG, DS, XZ, RJ, ML, DZ, SC, YY, LZ, and LP helped to prepare the manuscript. All authors have read and approved the final manuscript for publication.

## Funding

This work was supported by the China Agriculture Research System of MOF and MARA, and the Sichuan Veterinary Medicine and Drug Innovation Group of China Agricultural Research System (SCCXTD-2020-18).

## Conflict of interest

The authors declare that the research was conducted in the absence of any commercial or financial relationships that could be construed as a potential conflict of interest.

## Publisher’s note

All claims expressed in this article are solely those of the authors and do not necessarily represent those of their affiliated organizations, or those of the publisher, the editors and the reviewers. Any product that may be evaluated in this article, or claim that may be made by its manufacturer, is not guaranteed or endorsed by the publisher.
