# Supplementary material for: Specific DNAzymes cleave the 300–618 nt of 5′UTR to inhibit DHAV-1 translation and replication
Source: Front Microbiol. 2022 Dec 12;13:1064612. doi: 10.3389/fmicb.2022.1064612 (PMC9791187; doi:10.3389/fmicb.2022.1064612)
Supplement: Supplementary file 1 [file Data_Sheet_1.zip › Figure S1/Figure S1.docx]

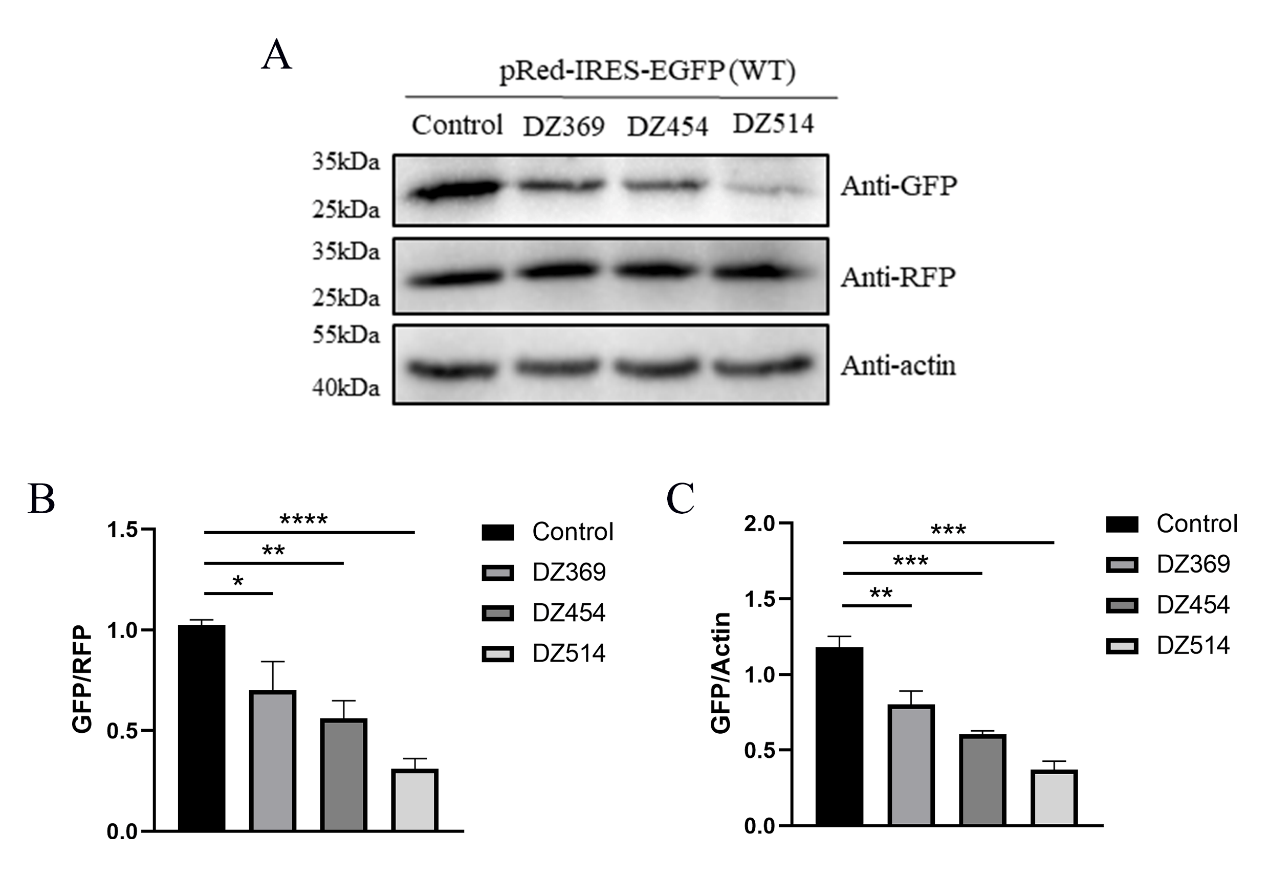


**Fig.** **S1 DNAzymes inhibit the translation mediated by the 300-618nt of DHAV-1 5’UTR in DF-1.** (A) pRed-IRES-EGFP was cotransfected with DZ369, DZ454, DZ514 or DZ000 (as the control group) in DF-1, and cells were harvested 48 h later and analysed by immunoblotting with the indicated antibodies. (B, C) Band intensity was quantified with ImageJ software, and RFP or β-actin was used as the mandatory control or loading control. The ratio of GFP to RFP or GFP to β-actin was normalized to the respective levels in the control group. Differences between two groups were analysed by Student’s t test and were considered significant when **P*<0.05, ***P*<0.01, ****P*<0.001, or *****P*<0.0001.
