# Supplementary figures and images for: Specific DNAzymes cleave the 300–618 nt of 5′UTR to inhibit DHAV-1 translation and replication
Source: Front Microbiol. 2022 Dec 12;13:1064612. doi: 10.3389/fmicb.2022.1064612 (PMC9791187; doi:10.3389/fmicb.2022.1064612)

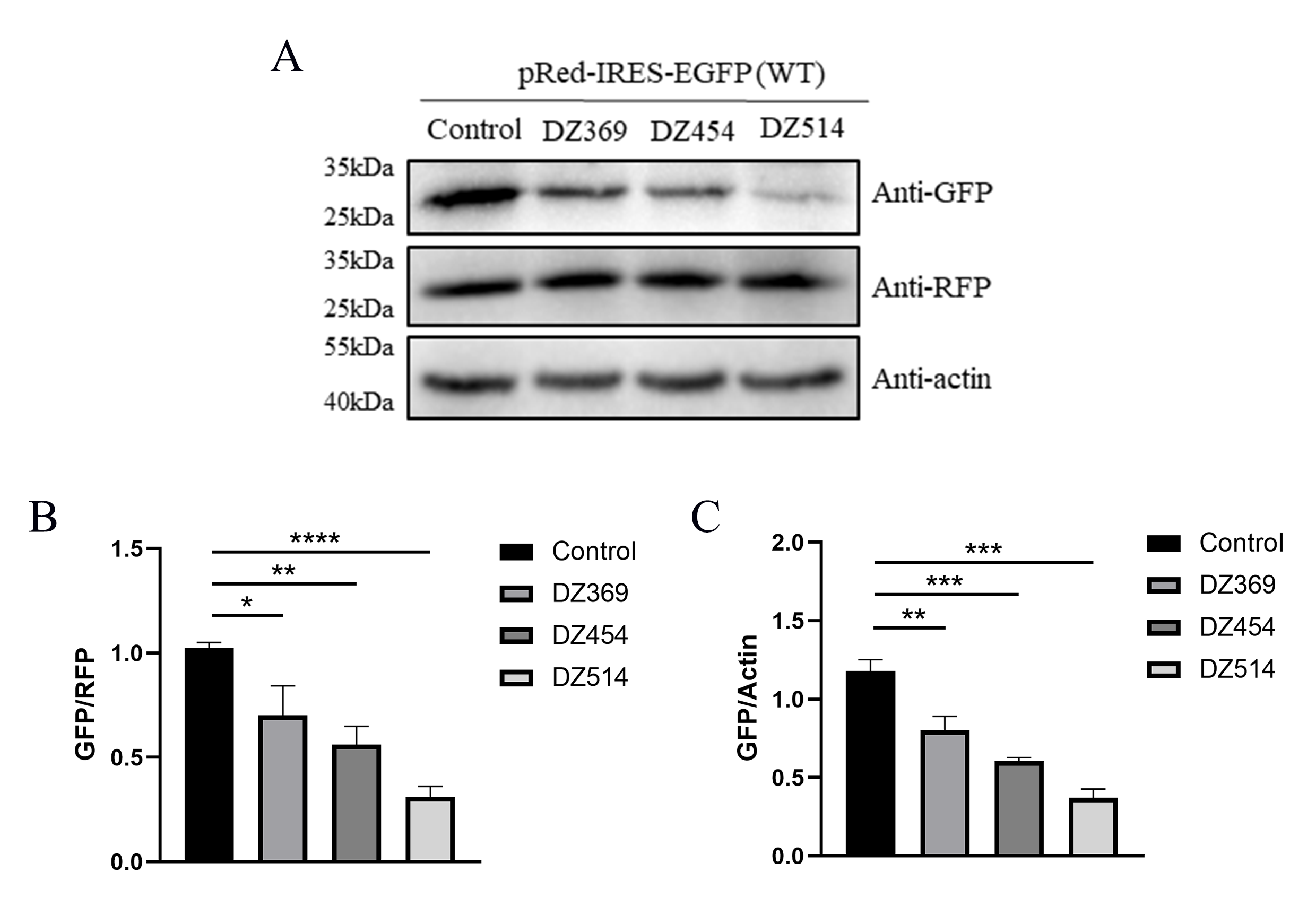

Supplement: Supplementary file 1 [file Data_Sheet_1.zip › Figure S1/Figure S1.tif]

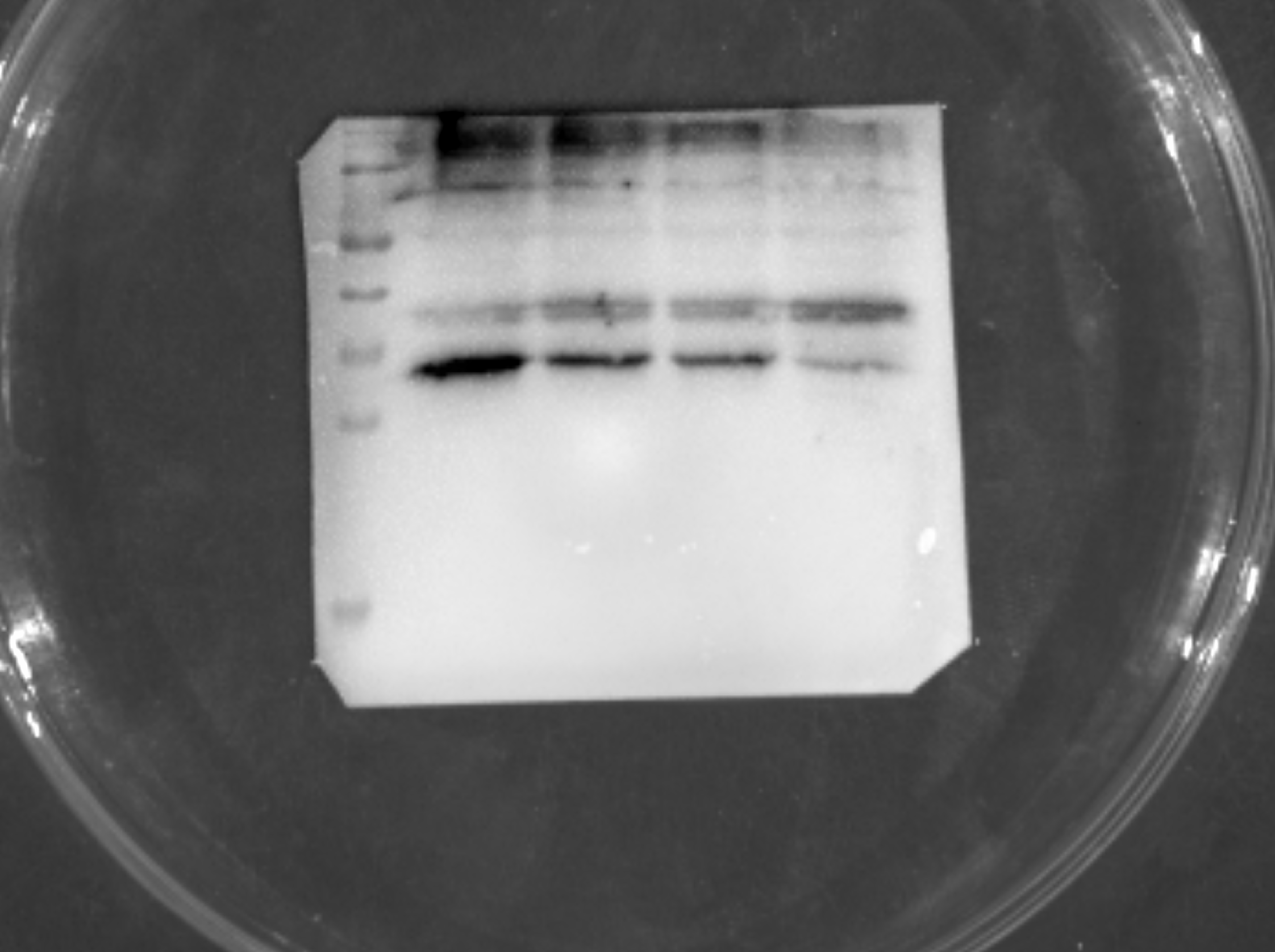

Supplement: Supplementary file 1 [file Data_Sheet_1.zip › Figure S1/S1 A/1/EGFP.tif]

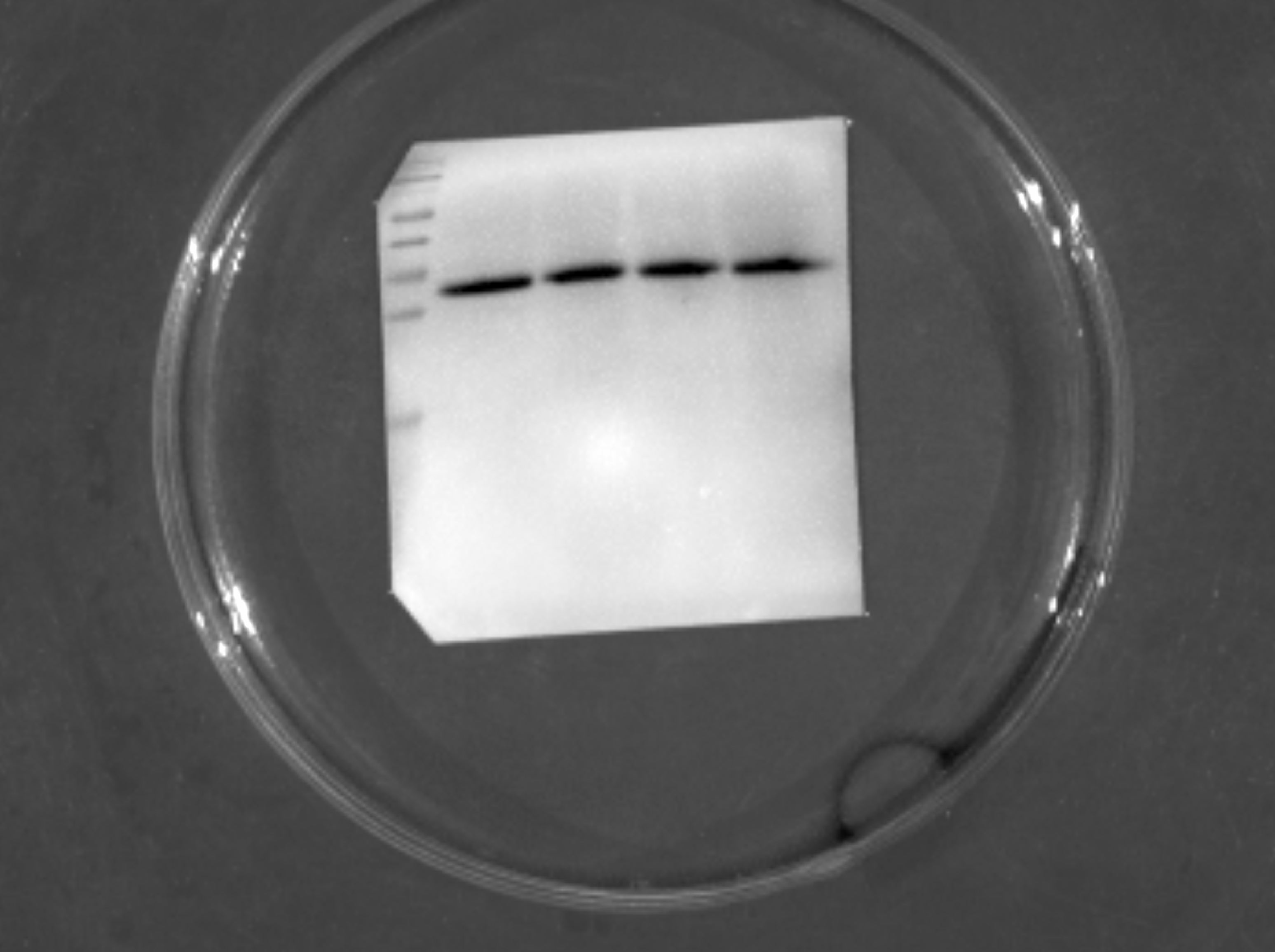

Supplement: Supplementary file 1 [file Data_Sheet_1.zip › Figure S1/S1 A/1/RFP.tif]

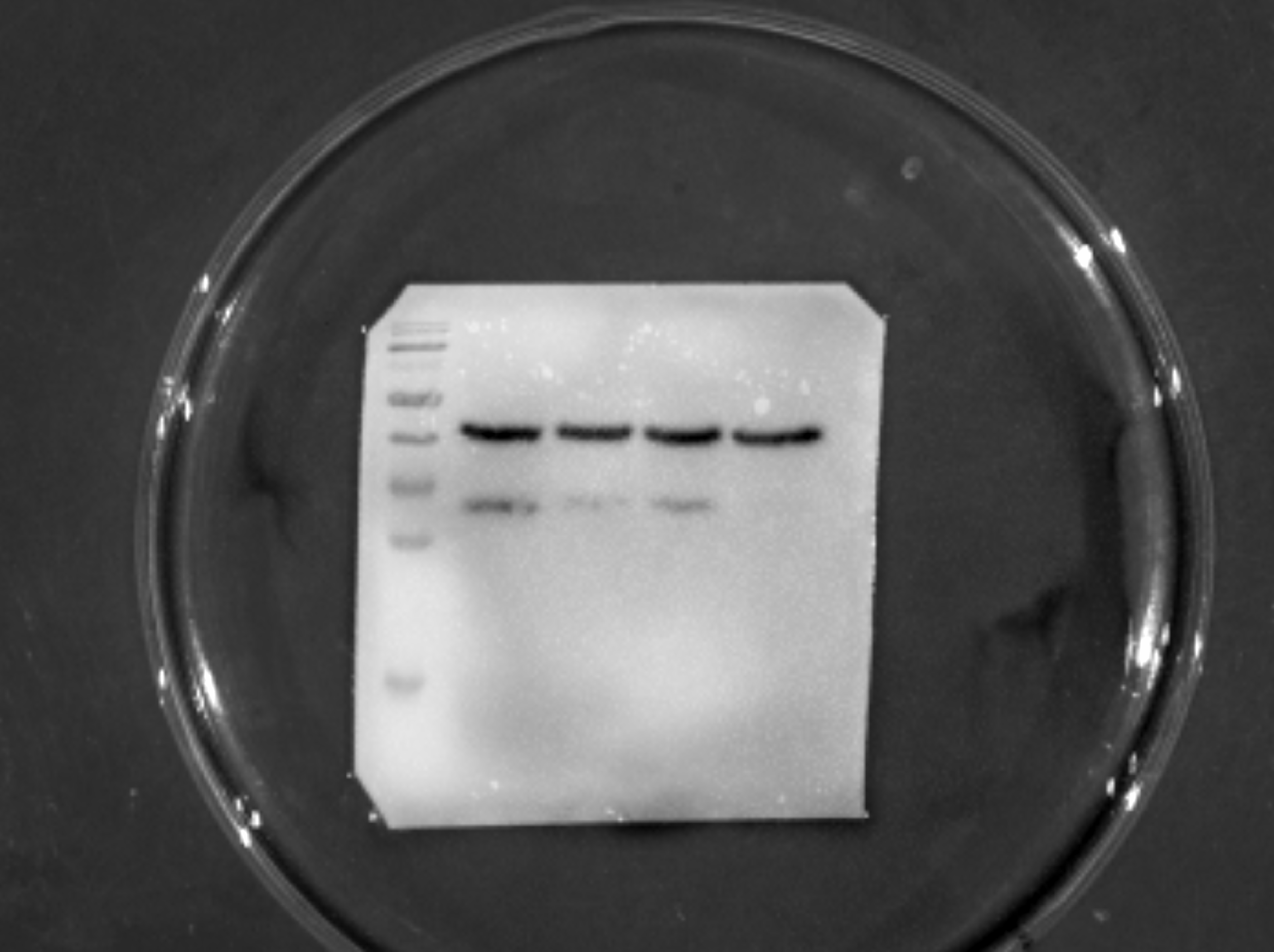

Supplement: Supplementary file 1 [file Data_Sheet_1.zip › Figure S1/S1 A/1/actin.tif]

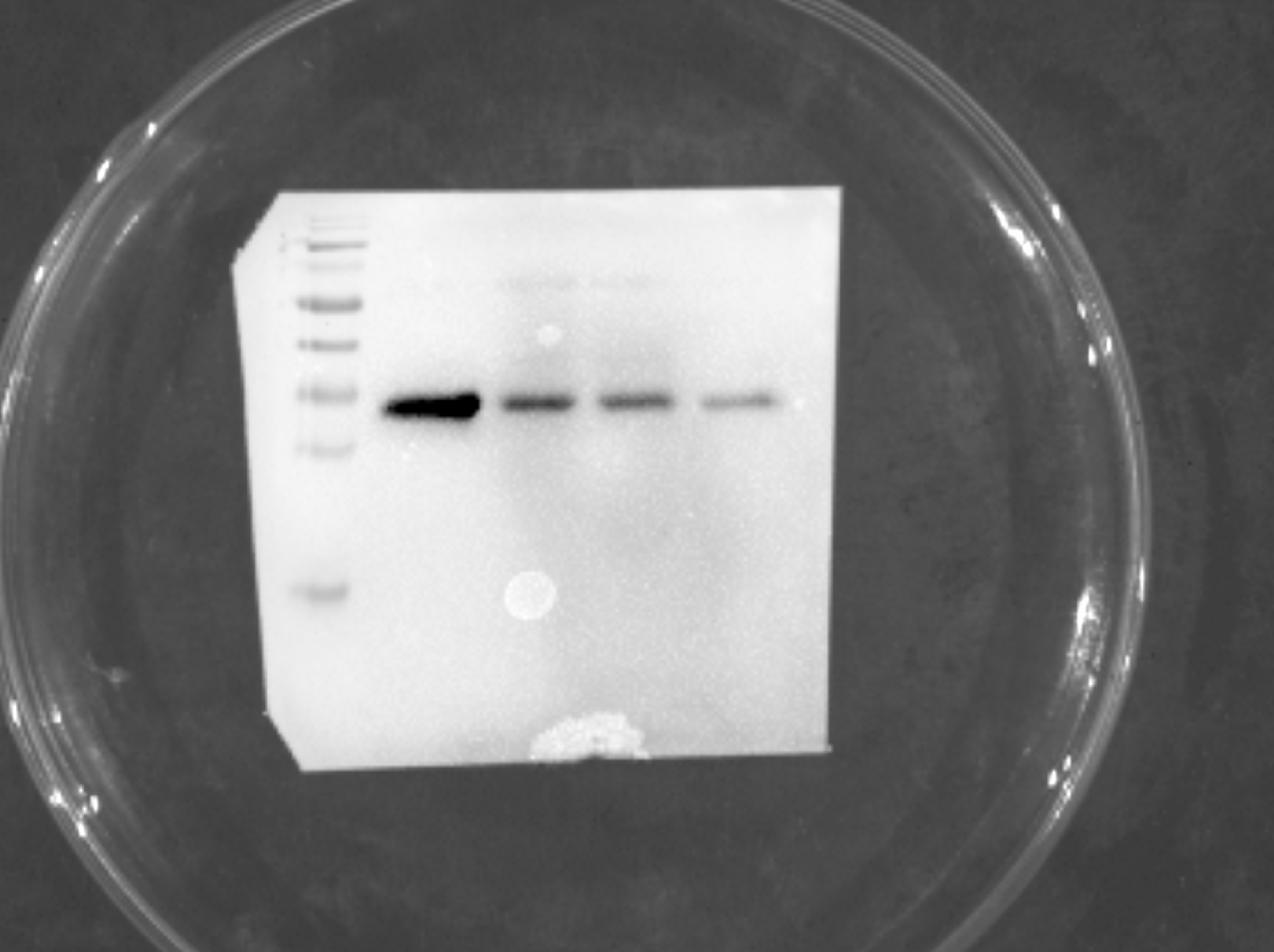

Supplement: Supplementary file 1 [file Data_Sheet_1.zip › Figure S1/S1 A/2/EGFP.tif]

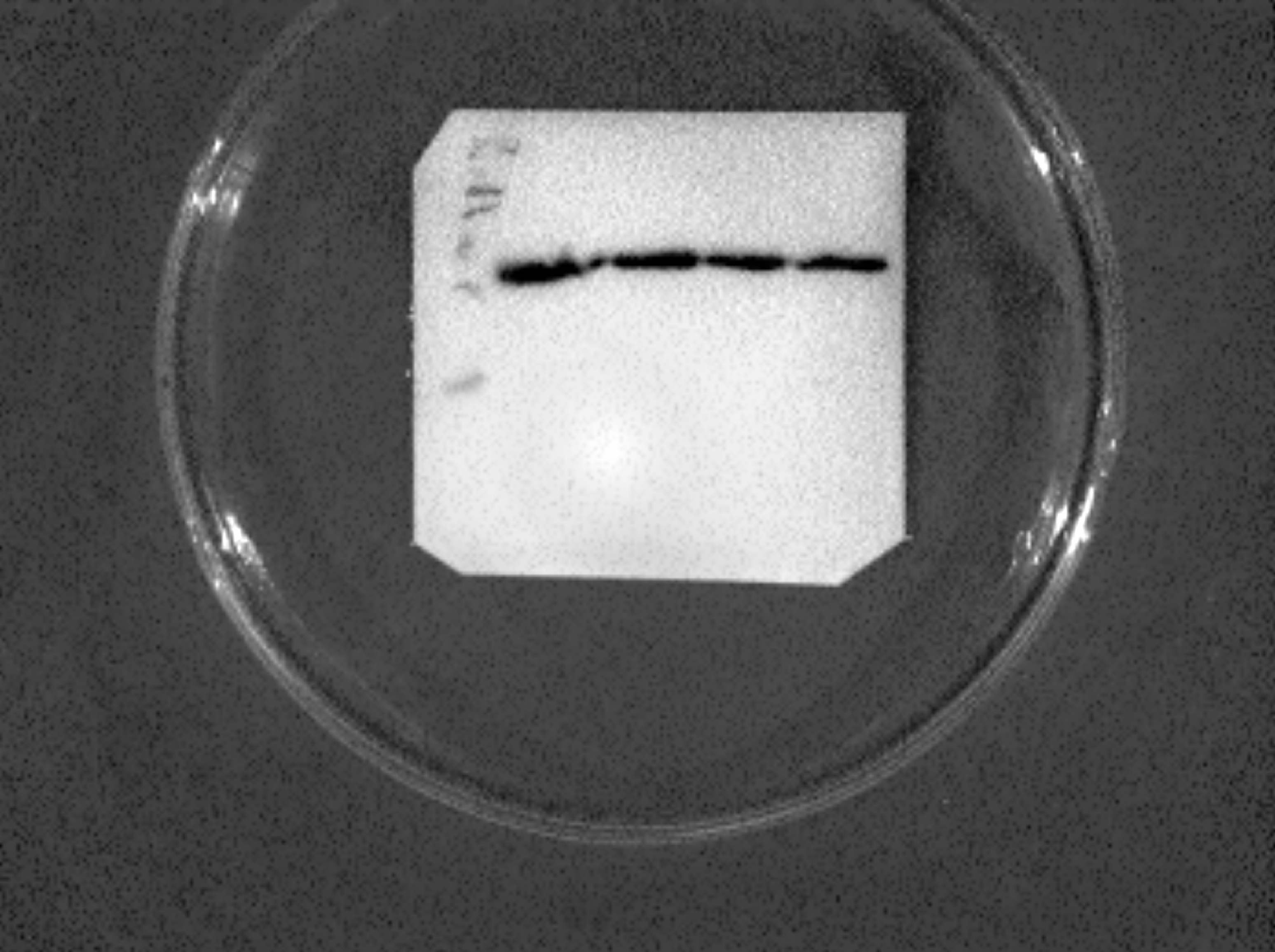

Supplement: Supplementary file 1 [file Data_Sheet_1.zip › Figure S1/S1 A/2/RFP.tif]

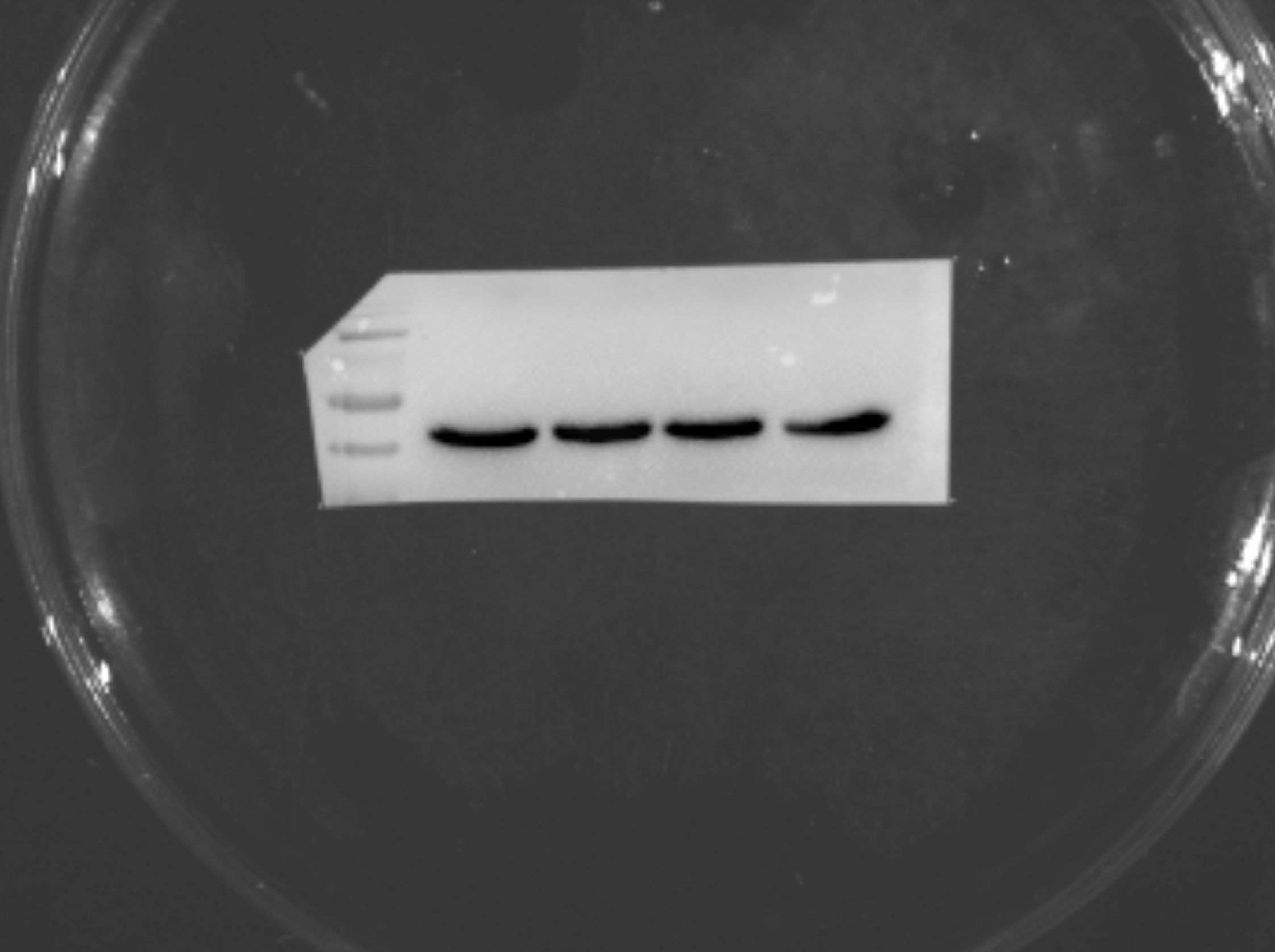

Supplement: Supplementary file 1 [file Data_Sheet_1.zip › Figure S1/S1 A/2/actin.tif]

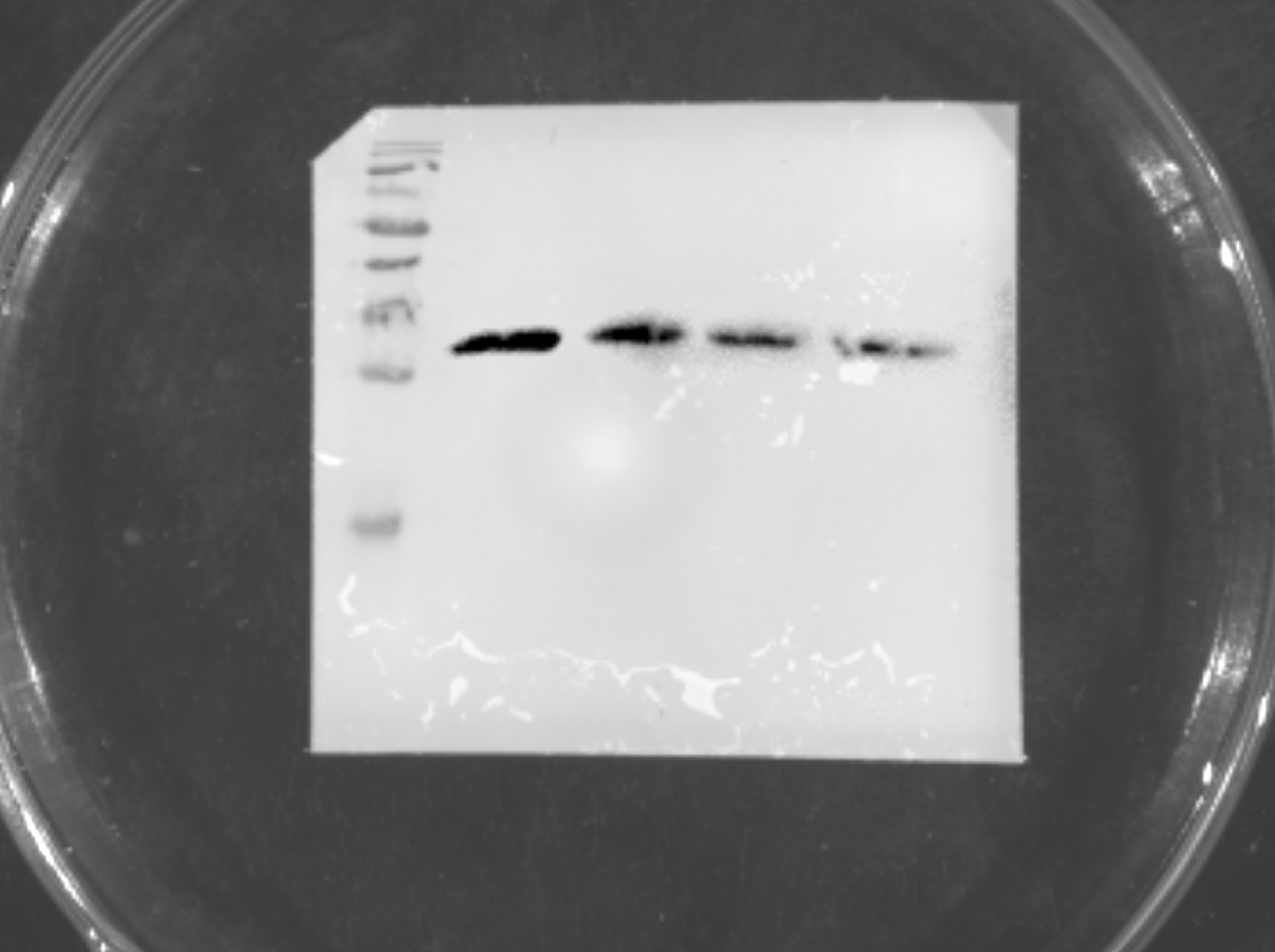

Supplement: Supplementary file 1 [file Data_Sheet_1.zip › Figure S1/S1 A/3/EGFP.tif]

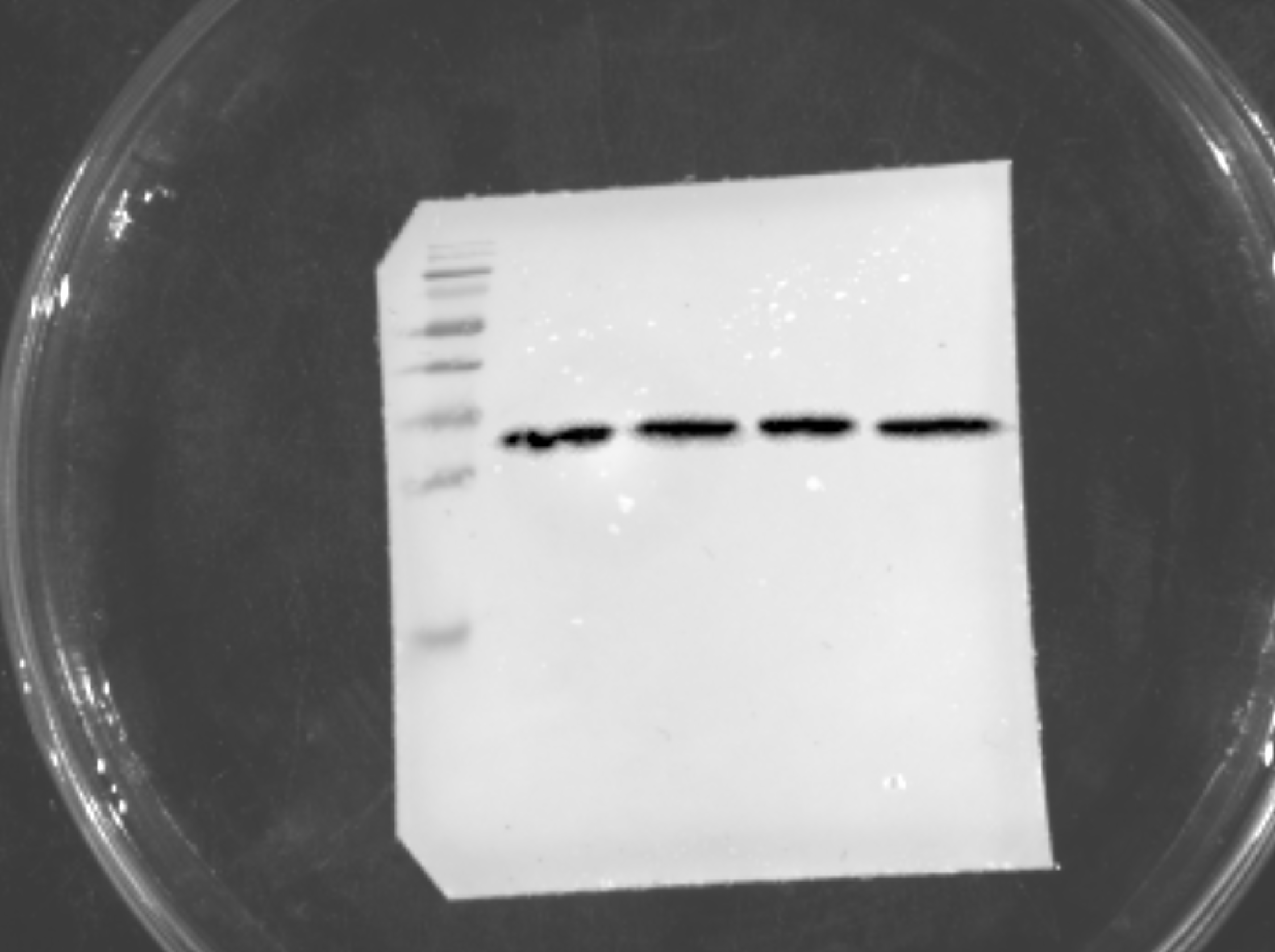

Supplement: Supplementary file 1 [file Data_Sheet_1.zip › Figure S1/S1 A/3/RFP.tif]

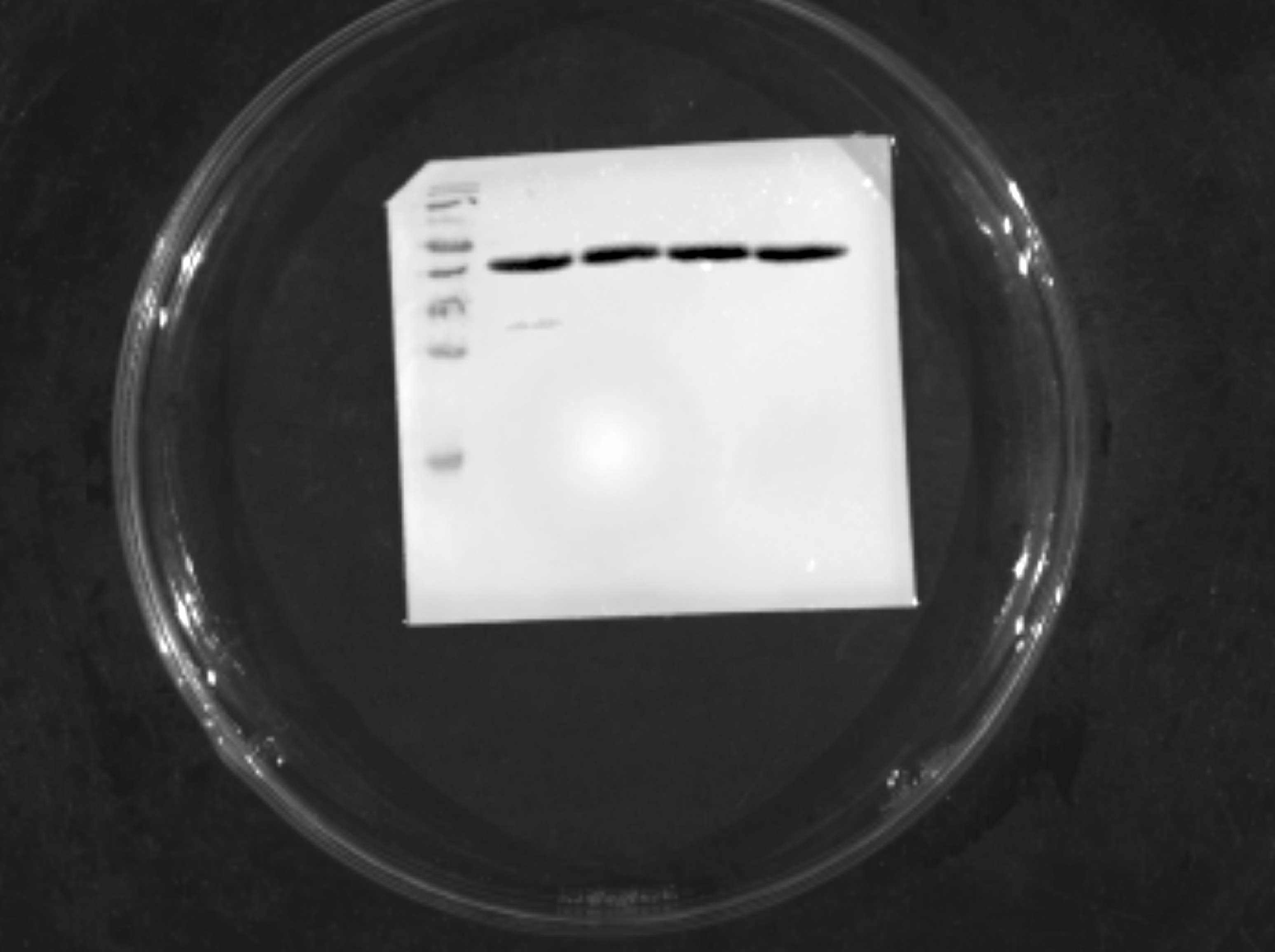

Supplement: Supplementary file 1 [file Data_Sheet_1.zip › Figure S1/S1 A/3/actin.tif]

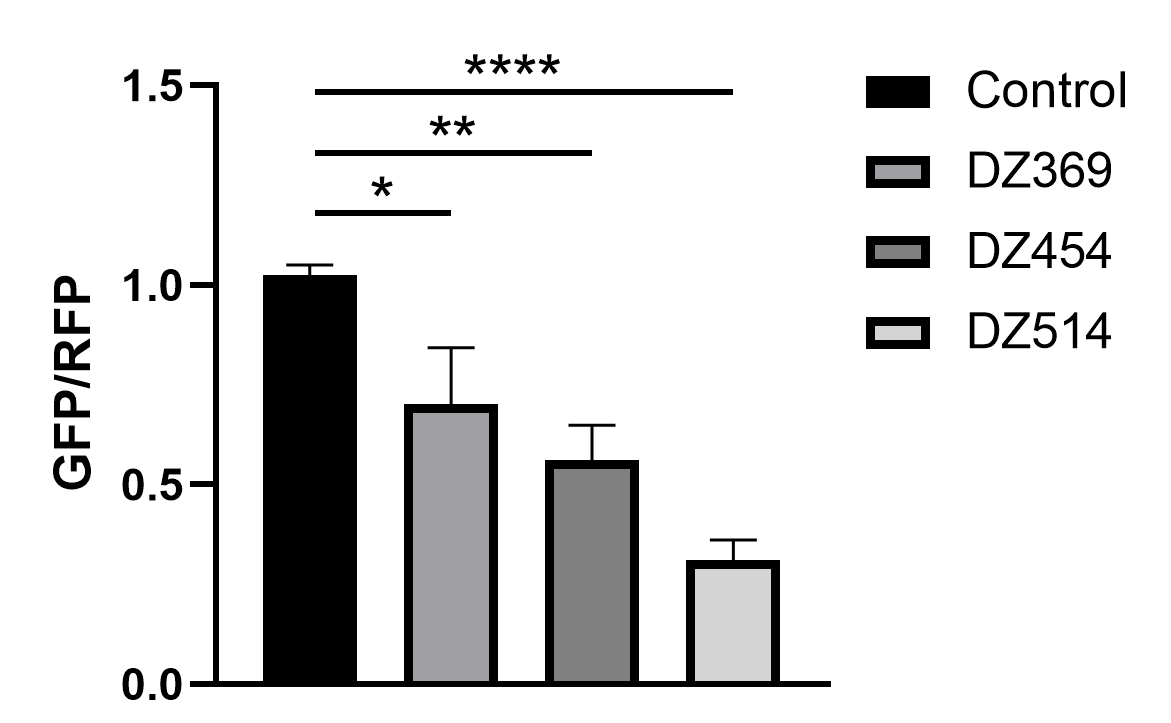

Supplement: Supplementary file 1 [file Data_Sheet_1.zip › Figure S1/S1 B/S1 B.tif]

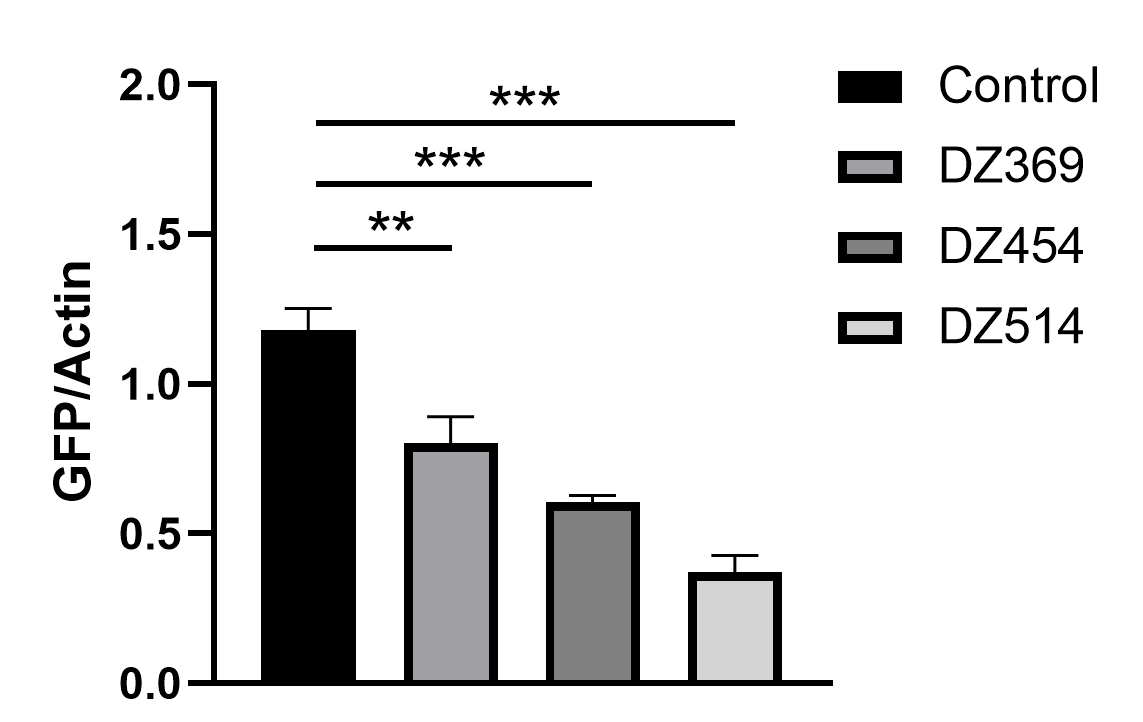

Supplement: Supplementary file 1 [file Data_Sheet_1.zip › Figure S1/S1 C/S1 C.tif]
